# High diversity in species, reproductive modes and distribution within the *Paramacrobiotus richtersi* complex (Eutardigrada, Macrobiotidae)

**DOI:** 10.1186/s40851-018-0113-z

**Published:** 2019-01-03

**Authors:** Roberto Guidetti, Michele Cesari, Roberto Bertolani, Tiziana Altiero, Lorena Rebecchi

**Affiliations:** 1 Department of Life Sciences, University of Modena and Reggio Emilia, Via Campi 213/D, 41125 Modena, Italy; 2 Department of Education and Humanities, University of Modena and Reggio Emilia, Via Allegri 9, 42121 Reggio Emilia, Italy; 3 Museo Civico di Storia Naturale of Verona, Lungadige Porta Vittoria 9, 37129 Verona, Italy

**Keywords:** 18S rRNA, Amphimixis, *cox1*, Cryptic species, Distribution, Karyotype, Parthenogenesis, Tardigrada

## Abstract

For many years, *Paramacrobiotus richtersi* was reported to consist of populations with different chromosome numbers and reproductive modes. To clarify the relationships among different populations, the type locality of the species (Clare Island, Ireland) and several Italian localities were sampled. Populations were investigated with an integrated approach, using morphological (LM, CLSM, SEM), morphometric, karyological, and molecular (18S rRNA, *cox1* genes) data. *Paramacrobiotus richtersi* was redescribed and a neotype designed from the Irish bisexual population. Animals of all populations had very similar qualitative and quantitative characters, apart from the absence of males and the presence of triploidy in some of them, whereas some differences were recorded in the egg shell. All populations examined had the same 18S haplotype, while 21 haplotypes were found in the *cox1* gene. In four cases, those qualitative characters were correlated with clear molecular (*cox1*) differences (genetic distance 14.6–21.8%). The integrative approach, which considered the morphological differences in the eggs, the reproductive biology and the wide genetic distances among putative species, led to the description of four new species (*Paramacrobiotus arduus* sp. n., *Paramacrobiotus celsus* sp. n., *Paramacrobiotus depressus* sp. n., *Paramacrobiotus spatialis* sp. n.) and two Unconfirmed Candidate Species (UCS) within the *P. richtersi* complex. *Paramacrobiotus fairbanksi*, the only ascertained parthenogenetic, triploid species, was redescribed and showed a wide distribution (Italy, Spain, Poland, Alaska), while the amphimictic species showed limited distributions. The difference in distribution between apomictic and amphimictic populations can be explained by the difference in the dispersal potentials associated with these two types of reproduction.

**Electronic supplementary material:**

The online version of this article (10.1186/s40851-018-0113-z) contains supplementary material, which is available to authorized users.

## Background

Molecular approaches are becoming increasingly essential in organismal systematics, phylogeny, biogeography and phylogeography. This is particularly true for the taxonomy of meiofaunal metazoans, which often consist of taxa that are neglected and difficult to identify due to their small sizes and low numbers of morphological characters. Cryptic species (i.e., two or more species classified as a single nominal species due to indistinguishable morphology [1]) have been found in several phyla of these kinds of organisms (e.g. polychaetes, flatworm, rotifers, gastrotrichs, etc. [2–7]). The discovery of cryptic diversity has profound implications for evolutionary theories [1], but the mechanisms of cryptic speciation in most taxa are unknown.

The presence of cryptic tardigrade species, evidenced by molecular methods, has also been reported in the literature [8–17]. In fact, in tardigrades genetic diversity within the same morphotype was observed before the use of DNA barcoding. Studies on allozymes in bisexual and unisexual populations attributed to the same morphospecies, *Richtersius coronifer* [18], showed a difference of 28% in the total genetic variation between them [19]. A recent study based on morphological and molecular approaches revealed that these two *Richtersius* populations belong to two different cryptic species [13]. Before the use of molecular approaches, several cases of different “cytotypes” of the same morphospecies were identified in several genera of eutardigrades (for a review, see [20]). These were often characterized by different chromosome numbers (namely diploid and polyploid) and/or a different reproductive mode (amphimixis vs parthenogenesis).

This kind of intriguing pattern is also detected in *Paramacrobiotus richtersi* (Murray, 1911) [21] (Eutardigrada, Macrobiotidae), a species previously considered widespread or cosmopolitan [22, 23], although its actual distribution is controversial [24]. *Paramacrobiotus richtersi* was reported to consist of populations with different chromosome numbers and reproductive modes [25, 26], but very probably it has to be considered the nominal species for a species complex (the *richtersi* group) requiring careful examination [27]. An updated and precise description of *P. richtersi* is needed to try to solve the taxonomy of the *richtersi* group and to better define the genus *Paramacrobiotus,* for which *P. richtersi* represents the type species. The original type material of *P. richtersi* should be present in the main repository of James Murray’s permanent slides, located in the Royal Scottish Museum of Edinburgh (Scotland, UK). However, in the catalogue of Tardigrada collection of the Museum [28], slides of *Macrobiotus richtersii* (the original species name) are not reported. To define a possible neotype of *P. richtersi* and to clarify the relationships among populations characterized by different ploidy and reproductive mode, we collected samples in the type locality in Ireland and in several Italian localities containing animals morphologically attributable to *P. richtersi*, and eggs of the “*richtersi* type” (according to the classification of Kaczmarek et al. [27]). These specimens were investigated with an integrative approach, studying morphology, karyology, reproductive biology, and DNA sequences. This combined approach allowed for the detection of an unexpectedly high species diversity characterized by a complex of very similar species, discriminated for their *cox1* sequences, but for the most part exclusively differentiated and uniquely recognized by their egg shell characters and/or reproductive biology.

## Methods

**Fig. 1:**
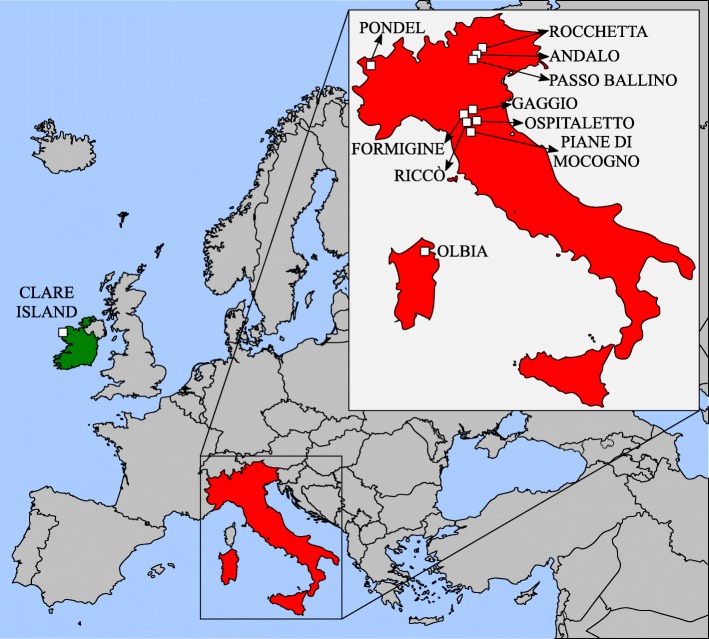
Sampling localities in Ireland and Italy

Twelve samples of leaf litter containing animals and eggs morphologically attributable to *P. richtersi* were collected in different Italian localities, whereas two samples were collected from the type locality (Kinnacorra, Clare Island, Mayo county; Ireland) (Table 1; Fig. 1). In the type locality, specimens corresponding to the original description of *P. richtersi* were found both in a moss sample (as in the original description) and in a turf sample. Only the turf sample was rich enough in animals to enable an integrative approach applying different methodologies. Eggs of this population were obtained from animals collected from the substrate and maintained in water at 15 °C in the laboratory until the oviposition.

**Table 1:** Analyzed populations, their sampling sites, sample number, kind of substrate, geographic coordinates, elevation, and type of analyses with number of analyzed specimens (LM, light microscopy; SEM, scanning electron microscopy, orcein, orcein staining; CLSM, confocal laser scanning microscopy; morphometry, morphometric data collection; *cox1* and 18S rRNA, molecular study of the corresponding gene)

Population (within brackets its abbreviation)	Sampling site	Sample number	Substrate	Geographical coordinates	a.s.l. (m)	Animals		Orcein	Eggs			Morphometry		DNA analysis	
						LM	SEM		LM	SEM	CLSM	Animals	Eggs	*cox1*	18S
Clare Island (CI)	Clare Island (Co. Mayo, Ireland)	C2714	turf	53°N 48.447 9°W 56.629	0	13	2	3	9	3		10	9	3	1
Clare Island (CI-m)	Clare Island (Co. Mayo, Ireland)	C2723	moss	53°N 48.447 9°W 56.629	0	5									
Rocchetta (Ro)	Rocchetta (Trento, Italy)	C2690	leaf litter	46°N 14.113011°E 03.463	350	10		24	29	7	1	6	8	3	1
Andalo (An)	Andalo (Trento, Italy)	C2762	leaf litter	46°N 09.742010°E 59.455	1100	12		10	10					1	
Passo Ballino (PB)	Passo Ballino (Trento, Italy)	C2693	leaf litter	45°N 58.724010°E 49.374	731	21		6	10	4	3	9	9	3	1
Pondel (Po)	Pondel (Aosta, Italy)	C2701	leaf litter	45°N 40.522007°E 13.344	890	16		7	7	7	2	9	9	2	1
Gaggio (Ga)	Gaggio (Modena, Italy)	C2698	leaf litter	44°N 37.736011°E 02.234	31	11		37	9	1		7	8	3	1
Formigine (Fo)	Formigine (Modena, Italy)	C2680	leaf litter	44°N 34.253010°E50.892	86	244	6	162	22	3	1	10	8	4	1
Ospitaletto (OA)	Ospitaletto (Modena, Italy)	C2794	leaf litter	44°N 26.521010°E 53.207	467	7		5						1	
Ospitaletto (OB)	Ospitaletto (Modena, Italy)	C2793	leaf litter	44°N 26.349010°E53.125	504	3		63	12					1	
Riccò (Ri)	Riccò (Modena, Italy)	C2683	leaf litter	44°N 25.880 010E 50.464	462	15		56	15	6		8	9	4	1
Piane di Mocogno (PM)	Piane di Mocogno (Modena, Italy)	C2112	leaf litter	44°N 16.775010°E 40.133	1312	16		16	9	3		11	9	3	1
Prodo (Pr)	Prodo (Terni, Italy)	C2703	leaf litter	42°N 45.965012°E 13.653	404	19		3	4		2	9	8	3	1
Olbia (Ol)	Olbia (Olbia-Tempio, Sardinia, Italy)	C2702	leaf litter	40°N 49.919009°E 21.304	175	72		8	11	2		10	9	1	1
*P. fairbanksi*	*locus typicus*											8	10

For each sample, specimens (animals and eggs) were mounted on permanent slides in Faure-Berlese fluid (as voucher specimens; paragenophores sensu Pleijel et al. [29]).

Specimens from all samples (Table 1) were fixed in Carnoy fluid (methanol: acetic acid, 3: 1) and stained with acetic lactic orcein for gender identification and chromosome analysis (for methods see also [30–32]). For gender evaluation, animals were also observed in vivo up to the maximum magnification (100× oil objective).

Specimens mounted on slides (Table 1) were investigated via light microscopy (LM) using phase contrast (PhC) and differential interference contrast (DIC) up to the maximum magnification of the objectives (100× oil objective). Measurements of the diagnostic characters of eggs and animals were also collected using LM (DIC) with a 40× objective and a 16× Leizt ocular micrometer. The sclerified structures of the animals were measured according to Pilato [33], and their *pt* indexes (the percent length of a structure with respect to the buccal tube length [33]) were computed. For the diameter of the buccal tube, the internal diameter at the level of the insertion of the stylet supports was considered. For the eggs, the process height, the internal diameter of the process base were measured, and processes for which at least half of the base diameter was present in an hemisphere were counted (the number of processes on the optical section was not considered, because being the processes with large bases only very few of them were present in any single optical section). Paratypes of *Paramacrobiotus fairbanksi* [34] (Table 1), provided by R. O. Schill (University of Stuttgart), were analysed and used for comparisons. Multivariate statistical analyses and principal component analysis (PCA) of the morphometric data were performed with the PAST software [35].

Eggs from five samples (Table 1) were investigated by confocal laser scanning microscopy (CLSM) with a TCS SP2 AOBS Spectral Confocal Scanner (Leica) mounted on a DM IRE2 inverted fluorescence microscope (Leica). Images were obtained through the use of an HeNe laser (405 nm/1.2 mW), taking advantage of the chitin autofluorescence of the sclerified structures [36, 37], and enhanced using Confocal Software Lite, version 2.61 (Leica).

Specimens from six Italian samples and from one Irish sample (Table 1) were prepared for scanning electron microscopy (SEM) following the protocol of Guidetti et al. [38], and observed with a SEM XL 40 (Philips). The SEM and CLSM were available at the ‘Centro Interdipartimentale Grandi Strumenti’ at the University of Modena and Reggio Emilia (Italy).

Molecular analysis was carried out on each population studied (Table 1). Total genomic DNA was extracted from single adult tardigrades, following the method described in [39]. All of the animals used for DNA extraction were first examined in vivo with LM using a 40× or 100× oil objective. A region of the nuclear ribosomal small subunit gene (18S rRNA) was amplified with the primer combination SSU F04 (5′-GCT TGT CTC AAA GAT TAA GCC-3′ [40]) and SSU R26 (3′-CAT TCT TGG CAA ATG CTT TCG-5′ [39]), following the protocol described in [41]. A fragment of the *cox1* mitochondrial gene was amplified following the protocol described in [39]. The amplicons were purified from gels using the Wizard Gel and PCR cleaning (Promega) kit. Both strands were subjected to the sequencing reaction using the Big Dye Terminator 1.1 kit (Applied Biosystems) and sequenced using an ABI Prism 3100 sequencer (Applied Biosystems). The nucleotide sequences of the newly analyzed specimens have been submitted to GenBank (acc. n.: MK040992-MK041032).

For the 18S gene analysis, 18S sequences from GenBank of other *Paramacrobiotus* specimens were included in the analysis (for GenBank acc. n. see Fig. 12). Nucleotide sequences were aligned with the MUSCLE algorithm, using default parameters implemented in MEGA6 [42]. The resulting alignment was inspected for accuracy by searching for software homology misinterpretations. The GBlocks program [43] was used for applying relaxed settings and parameters (values are as specified in [44]) and for aiming to discard uninformative regions of the alignment. Pairwise nucleotide sequence divergences between sequences were computed using p-distances by utilizing MEGA6.

For the *cox1* gene analysis, the chromatograms were checked for the presence of ambiguous bases; sequences were translated to amino acids by using the invertebrate mitochondrial code implemented in MEGA6 in order to check for the presence of stop codons and, therefore, of pseudogenes. Nucleotide sequences were aligned using the MUSCLE algorithm implemented in MEGA6 and were checked by visual inspection. For the molecular comparisons, *cox1* sequences from GenBank originating from other *Paramacrobiotus* specimens were included in the analysis (for GenBank acc. n. see Fig. 13)*.* Pairwise nucleotide sequence divergences between scored haplotypes were calculated using p-distances in MEGA6. Relationships between haplotypes were estimated using a parsimony network by applying the method described in [45], as implemented in TCS 1.21 [46] and visualized using tcsBU [47]. A 95% connection limit was employed as it has been suggested as a useful general tool in species assignments and discovery [48].

For the phylogenetic analyses, sequences of *Milnesium* (Apochela) and *Macrobiotus* (Parachela) specimens (for GenBank acc. n. see Fig. 12) were used as outgroups in the 18S dataset; a sequence of *Macrobiotus hufelandi* C.A.S. Schultze, 1834 [49] (GenBank acc. n. HQ876584) was used as an outgroup in the *cox1* dataset. A Bayesian inference (BI) phylogram was computed for both the 18S and *cox1* datasets using the program MrBayes 3.2 [50]. Best fitting model evaluations were performed taking into account the Akaike Information Criterion and Bayes Information Criterion (jModeltest 2 [51]) which identified the GTR + Γ model as the most suitable one. Two independent runs, each consisting of four Metropolis-coupled Markov chains using the Monte Carlo method, were launched for 5 × 10^6^ generations; trees were sampled every 100 generations. The convergence of runs was assessed by tracking the average standard deviation of split frequencies between runs and by plotting the log likelihood of sampled trees in Tracer v1.5 [52]; the first 500,000 sampled generations were discarded as burn-in. The analyses were run three times, all of which yielded identical topologies. A maximum likelihood (ML) analysis was also performed for both gene datasets, using the program RAxML v7.2.4 [53], with 1000 bootstrap replicates with rapid bootstrapping and a subsequent ML search with the GTR + Γ model.

The presence of putative species was inferred from *cox1* dataset by using the Poisson Tree Process (PTP), a coalescent-based species delimitation method that uses non-ultrametric gene trees as input [54] and heuristic algorithms to identify speciation events relative to numbers of substitutions. The starting gene tree was the ML tree computed as described above. The PTP method produces robust diversity estimates, even more that those estimated under the generalised mixed Yule coalescent model [55].

For possible further investigation, a fragment of each sample was desiccated and stored at − 20 °C, while several animals and eggs of each sample were preserved both in absolute ethanol and in Carnoy fluid at the BioBank of the EvoZoo Lab of the Department of Life Sciences, University of Modena and Reggio Emilia, Modena, Italy.

## Results

**Fig. 2:**
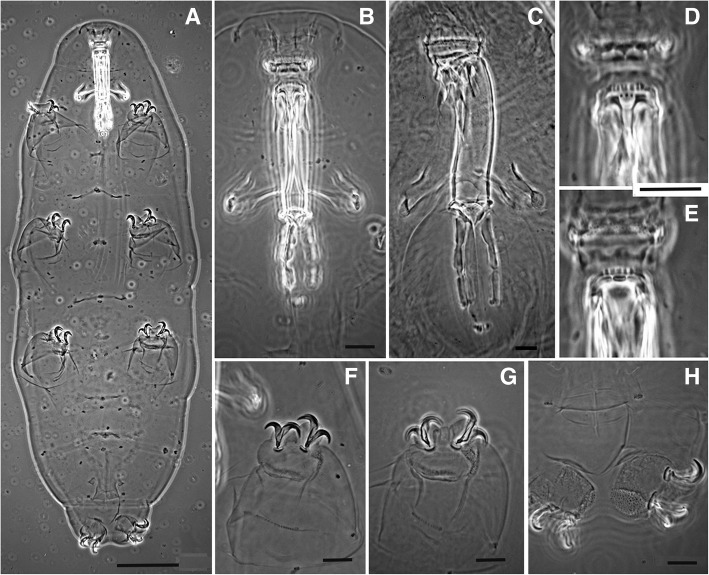
*Paramacrobiotus richtersi* (Clare Island) (PhC), neotype (**a**, **b**, **d-h**) and a specimen (**c**). **a** Habitus. **b** Buccal-pharyngeal apparatus (ventral). **c** Buccal-pharyngeal apparatus (ventrolateral) **d** Buccal armature (ventral view). **e** Buccal armature (dorsal view). **f** Claws of the first pair of legs. **g** Claws of the third pair of legs **h** Claws of the fourth pair of legs. Bars: **a** = 50 μm, **b**-**f** = 10 μm

**Fig. 3:**
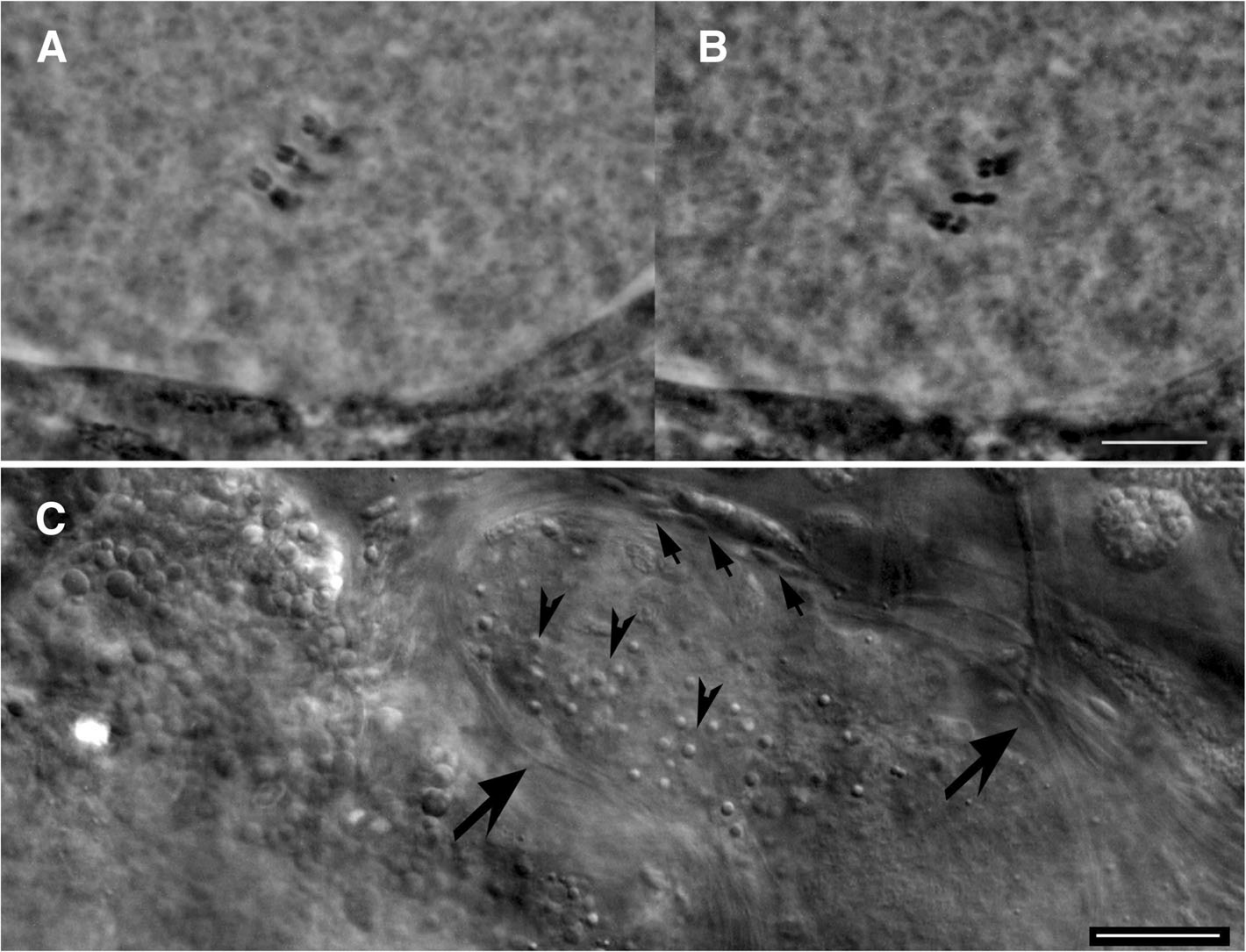
*Paramacrobiotus richtersi* from type locality (Clare Island). **a** and **b** Six bivalents in an oocyte in different focal planes (orcein; PhC). **c** Testis in vivo with spermatids (arrowheads) and spermatozoa (arrows) (DIC). Bars = 10 μm

The lack of *P. richtersi* type material necessitated the designation of a neotype and a re-description of the species (see Taxonomic account). The neotype is a male (Fig. 2) extracted together with other animals and eggs from a sample of turf (CI; Table 1) from the type locality of the species (Ireland). This male belongs to a bisexual population characterized by six bivalents in the oocytes (Fig. 3a, b). Males have also been identified in vivo (Fig. 3c) in specimens from both samples collected from Clare Island.

### Qualitative and quantitative comparisons of animals and eggs between Irish and Italian populations

**Fig. 4:**
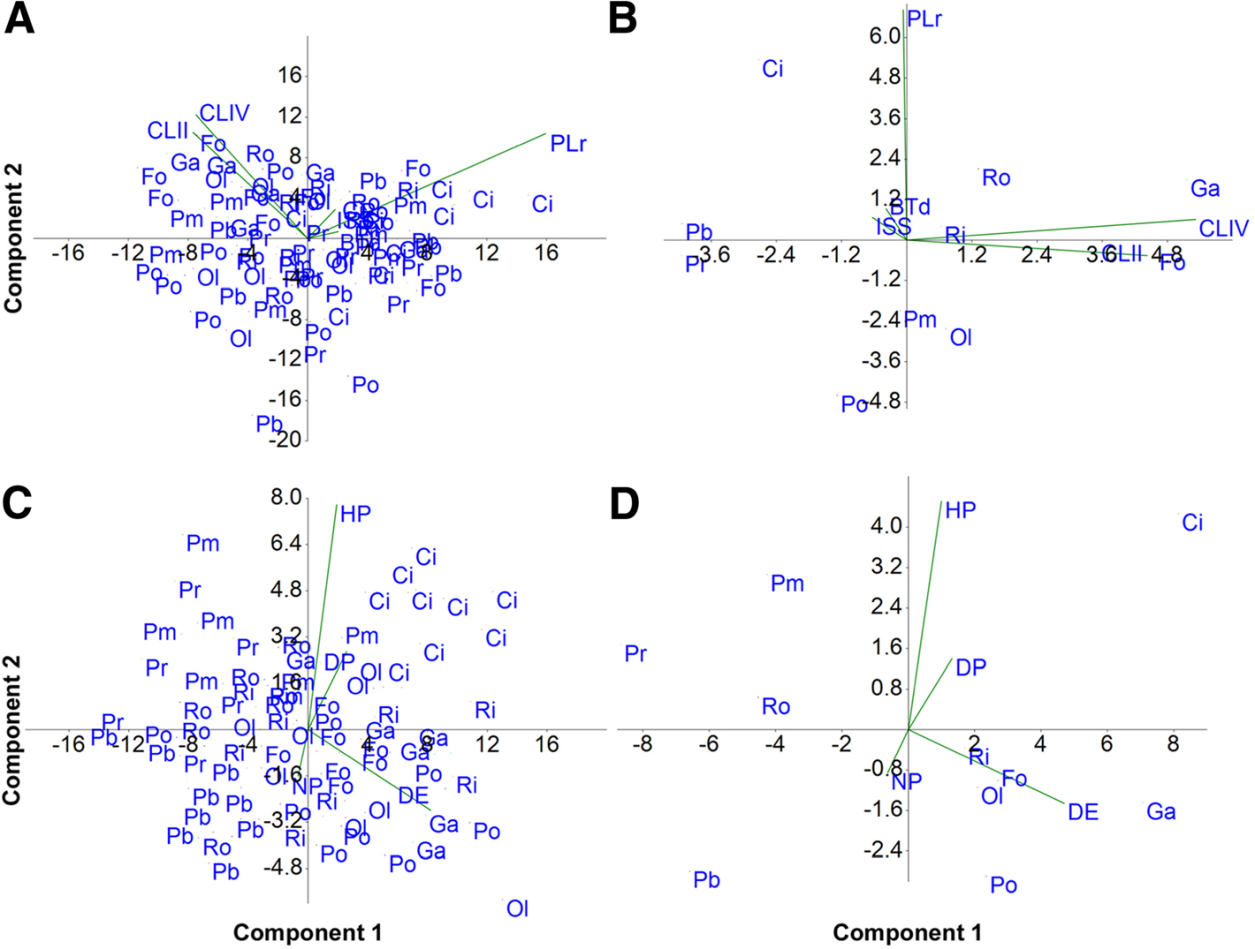
Principal Component Analysis (PCA) plots showing the multivariate variation among animals (**a** and **b**) and eggs (**c** and **d**) in terms of morphometric data. Vectors (green) indicate the direction and strength of each morphometric variable to the overall distribution. **a** Morphometric data of animals of the different populations. **b** Mean values of the morphometric data of animals in the different populations. **c** Morphometric data of eggs of the different populations. **d** Mean values of the morphometric of the data of the eggs in the different populations. Populations: CI = Clare Island; Fo = Formigine; Ga = Gaggio; PB = Passo Ballino; PM = Piane di Mocogno; Po = Pondel; Pr = Prodo; Ol = Olbia; Ri = Riccò; Ro = Rocchetta; for more information see Table 1. Morphometric data (according to Table 2, Additional file 1: Table S3): BTd = *pt* buccal tube diameter; ISS = *pt* insertion of the stylet support point; CLII = *pt* second claw pair; CLIV = *pt* fourth claw pair; PLr = *pt* macroplacoids row; DE = diameter of the egg; DP = diameter of the egg process; HP = height of the egg process; NP = number of processes per hemisphere. The first and second principal components explained the following variance, respectively: **a** 45.30 and 40.51%; **b** 53.70 and 39.68%; **c** 77.18 and 14.34%; **d** 81.81 and 14.39%

Animals from all of the Italian populations have qualitative and quantitative characters that are very similar both to each other and to those of the Irish population (Table 2; Additional file 1*:* Table S3). The populations can be distinguished only by their chromosome numbers, the presence or absence of males, and, consequently, by the mode of reproduction (see below). The maximum-minimum ranges of the *pt* indexes of the animals for all the measured characters and in all populations are overlapping (Table 2). Therefore, the populations are not distinguishable from one another using the morphometric characters related to the sclerified structures of the animals. Principal component analysis of the *pt* indexes of the sclerified structures does not show any defined separate cluster (Fig. 4a, b), confirming the data presented above. These results allow the conclusion that there is a *P. richtersi* complex characterized by species with extreme similarity in the morphologies of the sclerified structures of the animals.

**Table 2:** Morphometric data of the animals and eggs (measurements are in μm). n, number of analyzed specimens; totl, animal total length; btl, buccal tube length; btd, buccal tube diameter; *pt*, *pt* index; ssi, stylet support insertion on the buccal tube; mrl, macroplacoid row length; ecII, length of the external claw of the second leg pair; pcIV, length of the posterior claw of the hind leg; s.d., standard deviation

Population		Animal													Egg				
															n*.*	Egg	Process	proc. int.	n. proc.
		n.	totl	btl	btd	*pt-btd*	ssi	*pt-ssi*	mrl	*pt-mrl*	ecII	*pt-ecII*	pcIV	*pt-pcIV*		Diameter	Height	Diameter	hemisph.
Clare	Mean (s.d.)	10	652.8 (120.1)	49.7 (3.9)	8.2 (0.7)	16.5 (1.5)	40.3 (3.2)	81.1 (1.5)	32.8 (4.0)	65.8 (5.8)	13.9 (1.0)	28.1 (2.6)	14.8 (1.3)	29.9 (2.4)	9	74.0 (2.6)	20.2 (1.5)	18.8 (1.2)	15.4 (1.2)
Island	Range		385.0–770.0	41.6–55.1	7.3–9.8	14.1–18.9	33.4–43.6	78.4–83.3	24.8–38.9	56.9–74.5	11.9–15.5	25.1–33.4	12.3–16.5	25.6–33.7		70.3–78.2	17.1–22.1	17.2–21.2	12–18
Pondel	Mean (s.d.)	9	348.2 (58.1)	36.8 (4.9)	5.8 (1.0)	15.6 (0.9)	29.4 (4.4)	79.8 (1.9)	20.8 (4.3)	55.9 (4.6)	10.6 (1.7)	29.0 (3.9)	11.3 (2.0)	30.7 (4.0)	9	70.8 (6.0)	12.9 (1.8)	14.9 (2.3)	18.0 (1.3)
	Range		293.9–462.9	30.5–46.1	4.5–7.5	14.6–16.9	23.3–38.4	76.2–83.3	15.6–49.5	49.5–64.8	8.1–14.1	21.1–34.8	8.6–14.9	22.4–37.6		60.1–79.2	10.2–15.2	12.3–18.2	16–25
Rocchetta	Mean (s.d.)	6	619.3 (158.9)	53.8 (5.0)	9.6 (1.2)	17.8 (1.4)	43.2 (4.1)	80.4 (0.9)	33.5 (4.2)	62.2 (3.5)	16.5 (1.9)	30.7 (2.7)	17.6 (0.8)	33.0 (3.3)	8	63.6 (2.6)	15.3 (1.7)	13.6 82.3)	20.1 (0.9)
	Range		414.5–802.8	45.2–58.1	8.2–10.7	15.4–19.0	36.3–47.3	79.2–81.3	27.7–36.9	56.6–65.1	14.1–18.4	26.2–33.2	16.3–18.4	30.3–38.7		60.3–67.4	12.3–17.1	10.4–17.1	19–25
Passo	Mean (s.d.)	9	456 (155.5)	43.3 (6.3)	6.9 (1.5)	15.8 (1.4)	34.5 (4.8)	79.9 (3.9)	26.5 (5.6)	61.0 (6.6)	11.2 (2.0)	26.3 (2.7)	12.4 (1.6)	29.3 (4.6)	9	62.2 (3.0)	10.8 (1.2)	13.3 (0.9)	18.4 (1.6)
Ballino	Range		280.2–768.5	33.7–52.8	4.7–9.4	13.9–17.9	26.9–42.1	69.7–82.6	17.9–35.4	49.2–67.1	8.8–15.1	22.2–29.6	10.1–14.6	22.2–35.7		56.2–66.2	9.3–12.4	12.4–15.2	16–23
Piane di	Mean (s.d.)	11	535.2 (115.1)	46.9 (5.3)	7.2 (1.2)	15.3 (1.3)	37.7 (4.2)	80.3 (1.2)	27.5 (4.5)	58.5 (5.7)	14.2 (1.7)	30.3 (3.0)	14.4 (1.3)	31.2 (3.6)	9	62.8 (3.5)	16.8 (1.6)	15.4 (1.3)	18.1 (1.2)
Mocogno	Range		408.9–758.4	38.2–54.5	5.4–9.1	13.9–18.1	30.6–44.1	78.9–82.7	20.3–34.5	50.3–66-6	12.2–17.8	26.5–34.9	12.1–15.8	24.7–36.5		58.1–69.1	15.2–19.1	14.3–18.2	15–19
Formigine	Mean (s.d.)	10	515.4 (113.6)	44.7 (6.4)	6.9 (1.1)	15.5 (1.2)	36.0 (5.1)	80.5 (1.3)	26.9 (5.0)	60.0 (4.5)	14.6 (2.1)	33.1 (5.1)	15.6 (2.3)	35.1 (3.9)	8	70.3 (3.1)	14.1 (1.1)	16.1 (1.8)	16.9 (1.5)
	Range		325.1–751.1	32.9–57.6	5.3–9.2	14.0–17.4	26.4–46.2	77.9–82.5	18.4–36.5	54.5–69.1	10.7–16.9	21.3–39.7	10.5–17.9	28.2–40.3		65.4–75.1	13.2–16.2	15.2–20.4	15–23
Riccò	Mean (s.d.)	8	519.2 (45.2)	47.1 (2.5)	7.8 (0.6)	16.5 (0.6)	38.2 (2.0)	81.1 (0.5)	28.7 (2.1)	60.7 (1.5)	14.1 (0.9)	30.3 (0.5)	15.3 (1.2)	32.4 (1.2)	9	69.7 (5.0)	15.6 (2.1)	14.9 (3.0)	19.3 (1.0)
	Range		362.8–790.7	38.1–60.2	6.3–11.4	15.0–19.0	31.6–48.9	78.9–83.1	21.9–40.8	55.6–67.8	11.6–18.3	28.2–31.9	12.0–20.1	26.6–34.8		65.3–78.1	13.4–19.1	11.2–20.1	18–22
Gaggio	Mean (s.d.)	7	489.3 (118.1)	44.6 (4.9)	7.1 (1.1)	15.8 (1.1)	35.3 (4.0)	79.1 (1.9)	27.8 (4.9)	62.1 (4.8)	14.0 (1.2)	31.8 (4.2)	16.1 (1.4)	36.8 (3.8)	8	74.9 (3.3)	15.4 (1.2)	16.5 (1.9)	18.3 (2.0)
	Range		370.5–715.7	38.6–52.0	5.4–8.5	14.1–17.5	29.8–40.7	77.3–81.7	23.1–34.1	56.6–70.1	12.6–15.8	25.9–36.8	14.1–17.5	30.6–41.7		68.2–78.2	14.1–17.3	13.1–18.1	18–26
Prodo	Mean (s.d.)	9	456.5 (81.6)	45.5 (3.1)	7.8 (0.7)	17.1 (1.0)	36.9 (2.6)	81.1 (1.7)	27.2 (2.6)	59.9 (2.7)	12.2 (1.3)	26.9 (2.4)	13.1 (1.1)	29.0 (3.3)	8	59.1 (2.6)	14.7 (2.2)	13.4 (2.0)	18.2 (1.9)
	Range		344.7–610.0	38.6–49.4	6.7–8.9	15.1–18.4	30.8–39.9	78.2–84.4	21.8–30.3	54.2–63.2	10.0–14.2	22.2–30.6	11.9–14.9	25.9–37.1		55.3–62.3	12.1–18.3	10.4–16.3	16–21
Olbia	Mean (s.d.)	10	435.1 (73.5)	42.6 (4.7)	6.8 (1.2)	16.0 (1.8)	33.9 (4.0)	79.4 (1.9)	24.8 (4.4)	57.9 (5.7)	13.1 (2.1)	30.6 (3.0)	13.8 (2.4)	32.2 (3.4)	9	69.9 (6.0)	14.1 (2.2)	15.7 (1.5)	16.9 (1.2)
	Range		298.8–536.7	32.2–48.6	5.1–9.1	12.5–18.8	24.9–38.8	76.2–82.6	16.4–29.4	49.2–67.6	8.8–15.5	27.1–35.4	9.0–16.9	28.0–37.5		61.4–82.2	12.3–18.2	14.2–18.1	15–20
*P. fairbanksi*	Mean (s.d.)	8	452,3 (113.0)	54.3 (5.8)	10.2 (1.6)	18,8 (2.2)	42.8 (4.5)	78.7 (1.8)	29.9 (4.0)	55.0 (2.3)	14.8 (1.7)	27.2 (1.4)	15.9 (2.0)	29.2 (1.5)	10	71.9 (6.6)	12.5 (1.4)	14.7 (2.7)	19.2 (1.5)
paratypes	Range		254.0–597.0	42.6–59.4	6.9–11.9	16.3–23.5	33.7–47.5	75.0–80.8	21.8–33.7	51.2–57.9	11.9–16.8	25.4–29.8	11.9–17.3	26.9–31.4		62.4–83.2	10.9–14.9	10.9–20.8	17–22

**Fig. 5:**
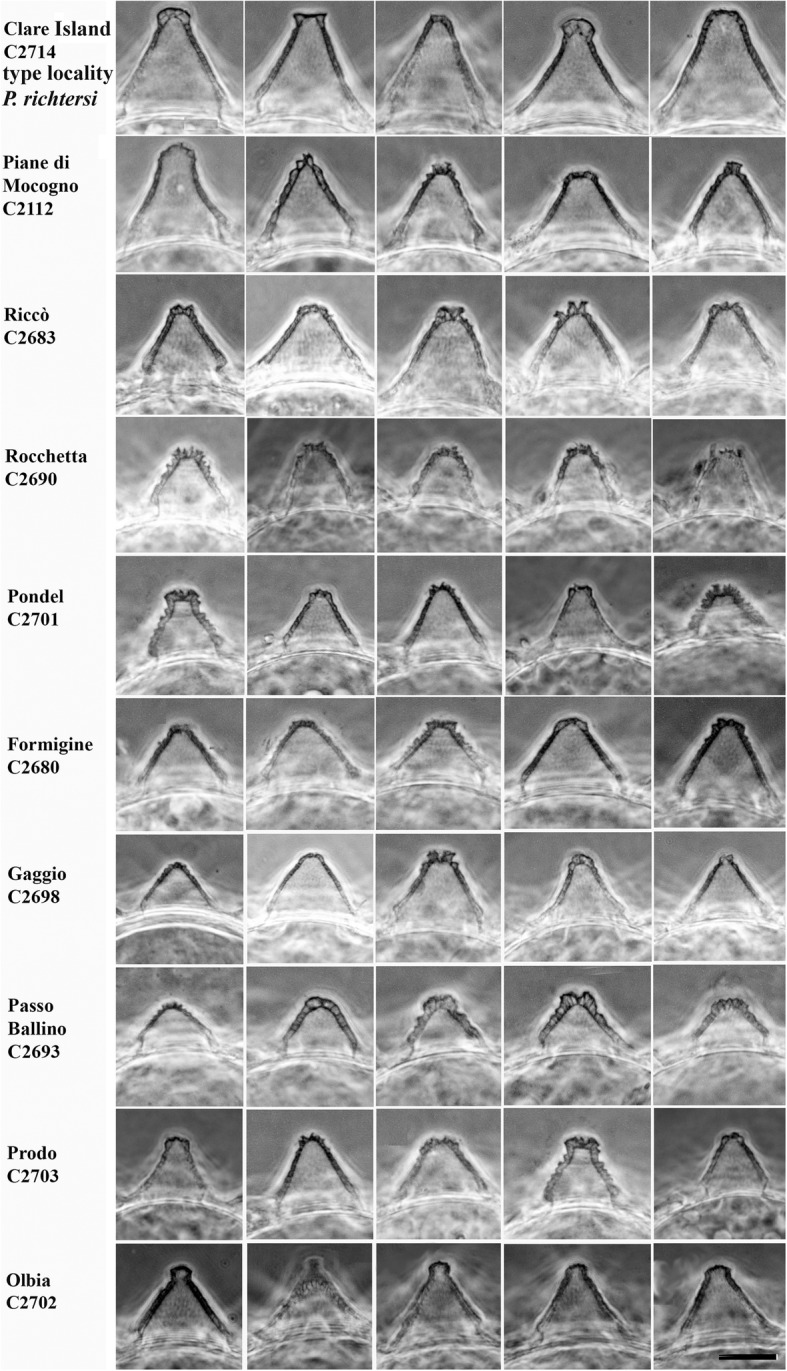
Variability of the egg process morphologies among and within the eggs of the studied populations (PhC). Bar = 10 μm (for all figures)

**Fig. 6:**
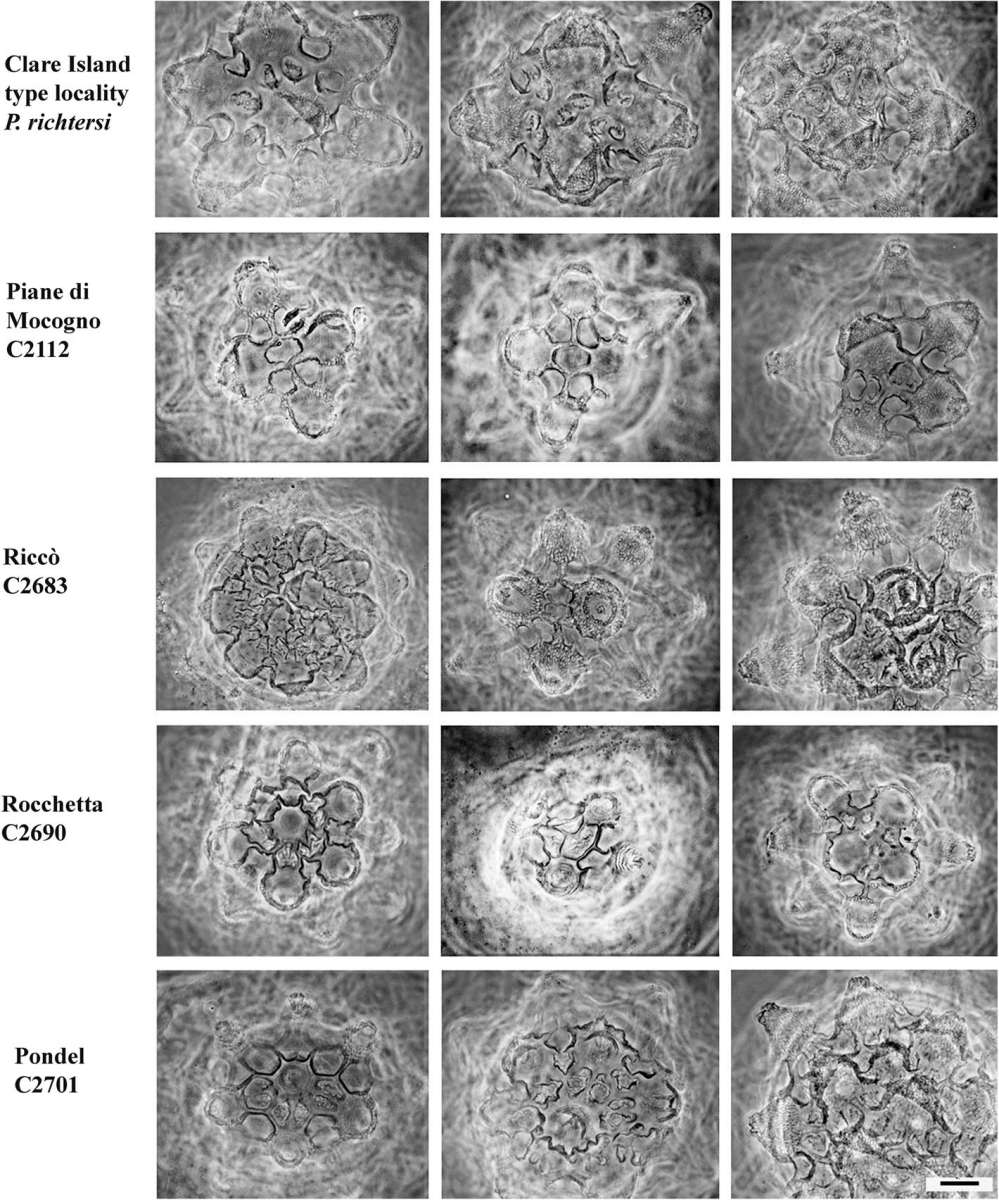
Areolae (around each process) variability in the egg shell among and within the Clare Is., Piane di Mocogno, Riccò, Rocchetta, Pondel populations (PhC). Bar = 10 μm (for all figures)

**Fig. 7:**
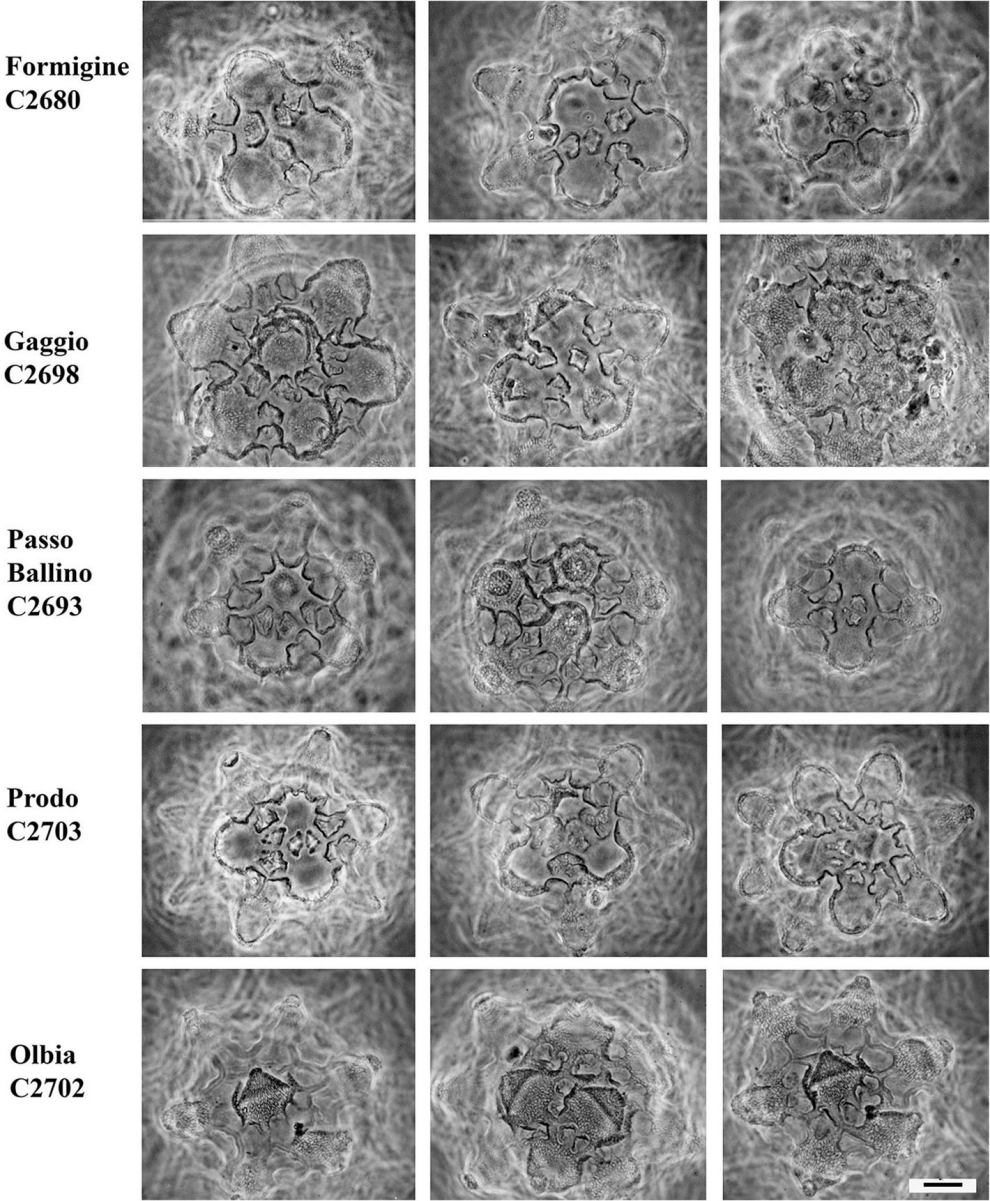
Areolae (around each process) variability in the egg shell among and within the Formigine, Gaggio, Passo Ballino, Prodo, Olbia populations (PhC). Bar = 10 μm (for all figures)

**Fig. 8:**
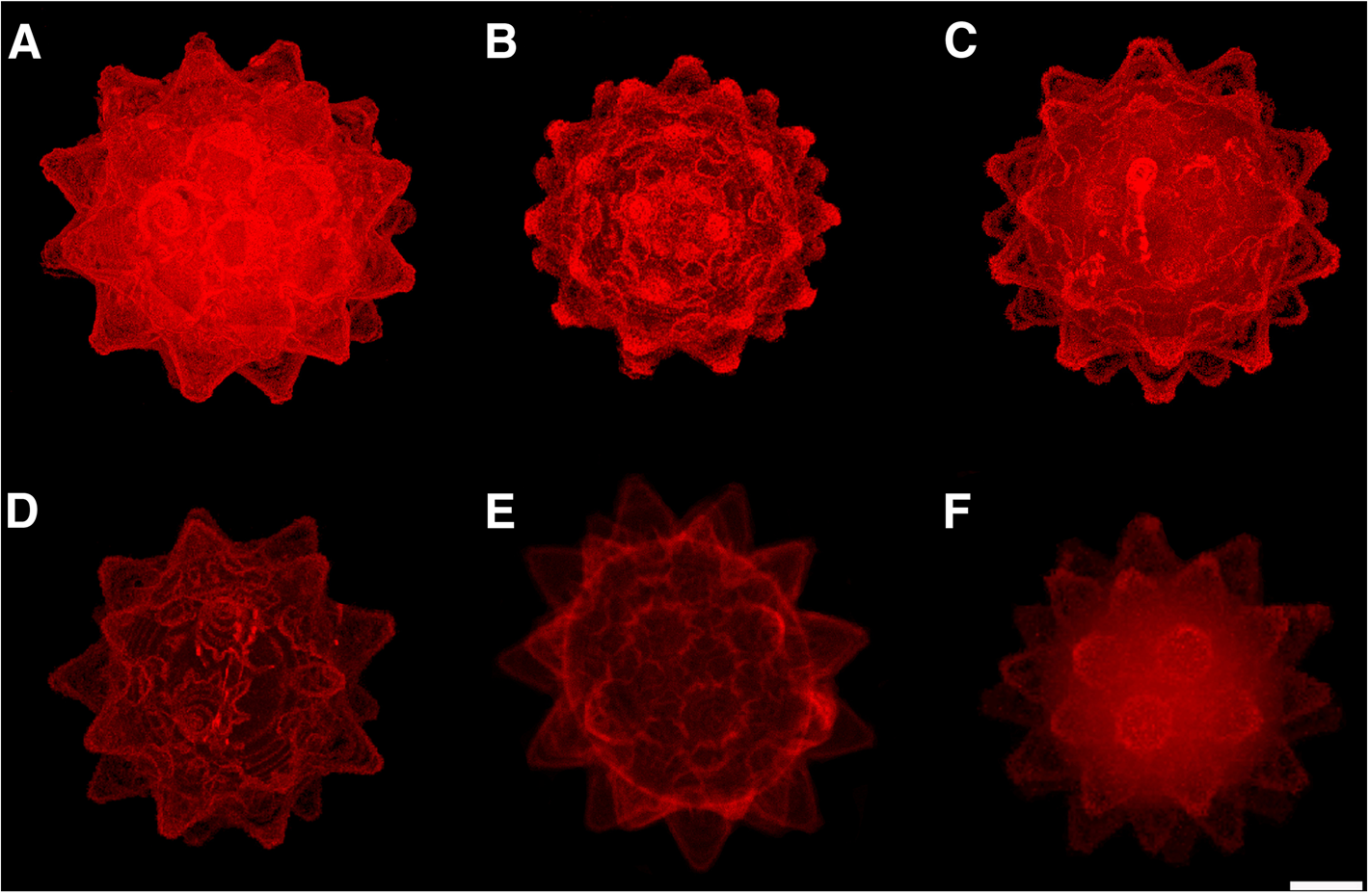
Eggs of the *Paramacrobiotus* populations (CLSM). **a** Formigine. **b** Passo Ballino. **c** Pondel (the buccal-pharyngeal apparatus of the embryo at the end of development is visible). **d** Prodo (the buccal-pharyngeal apparatus of the embryo at the end of development is visible). **e** Prodo. **f** Rocchetta. **a**-**d** Maximum projection, **e** and **f** Average projection. Bar = 20 μm (for all figures)

**Fig. 9:**
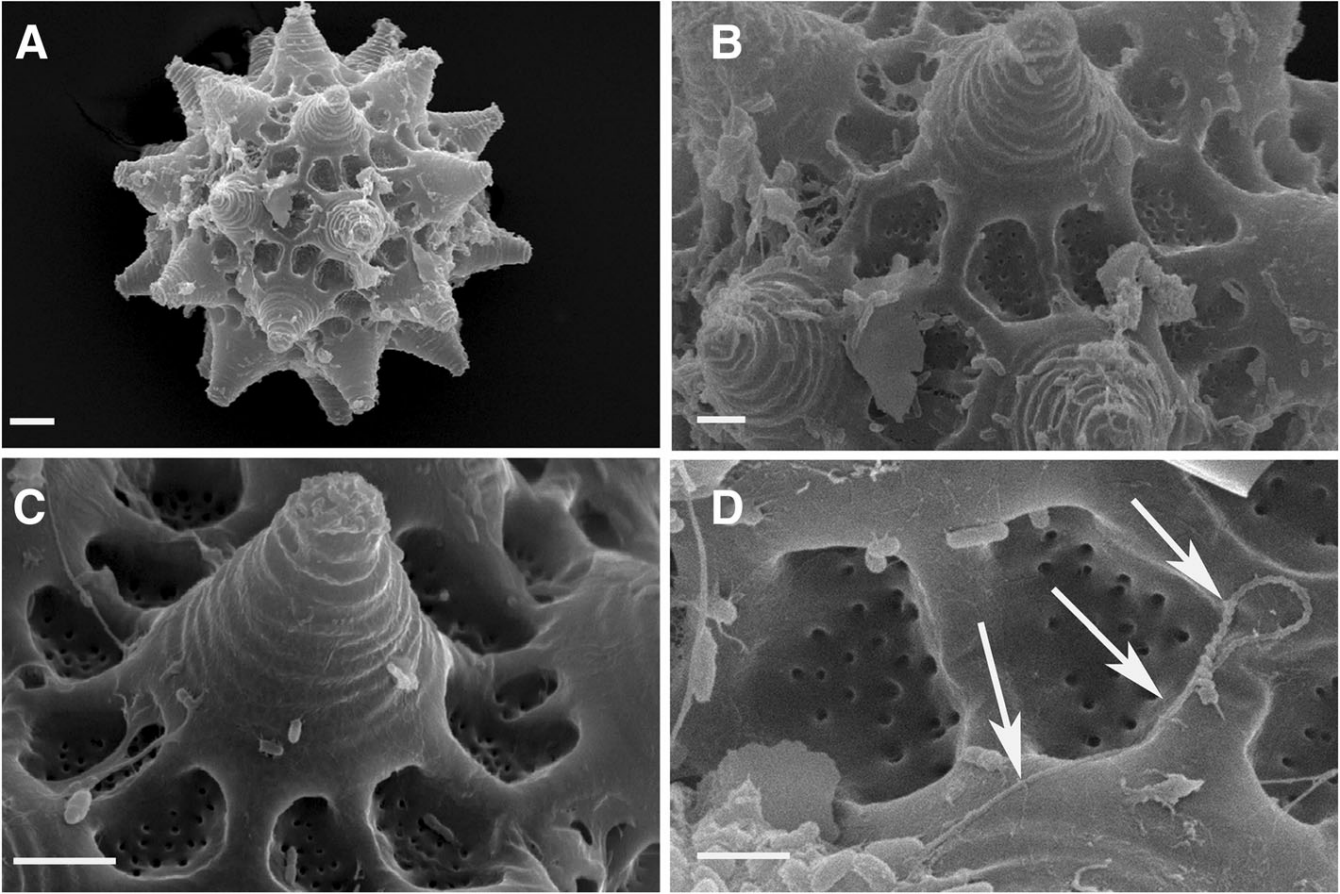
Eggs of *Paramacrobiotus richtersi* (SEM). **a** In toto*.* **b** Egg processes. **c** Egg process with areolae at the base. **d** Spermatozoon (arrows) on the egg shell. Bars: **a** = 10 μm, **b** and **c** = 5 μm, **d** = 2 μm

**Fig. 10:**
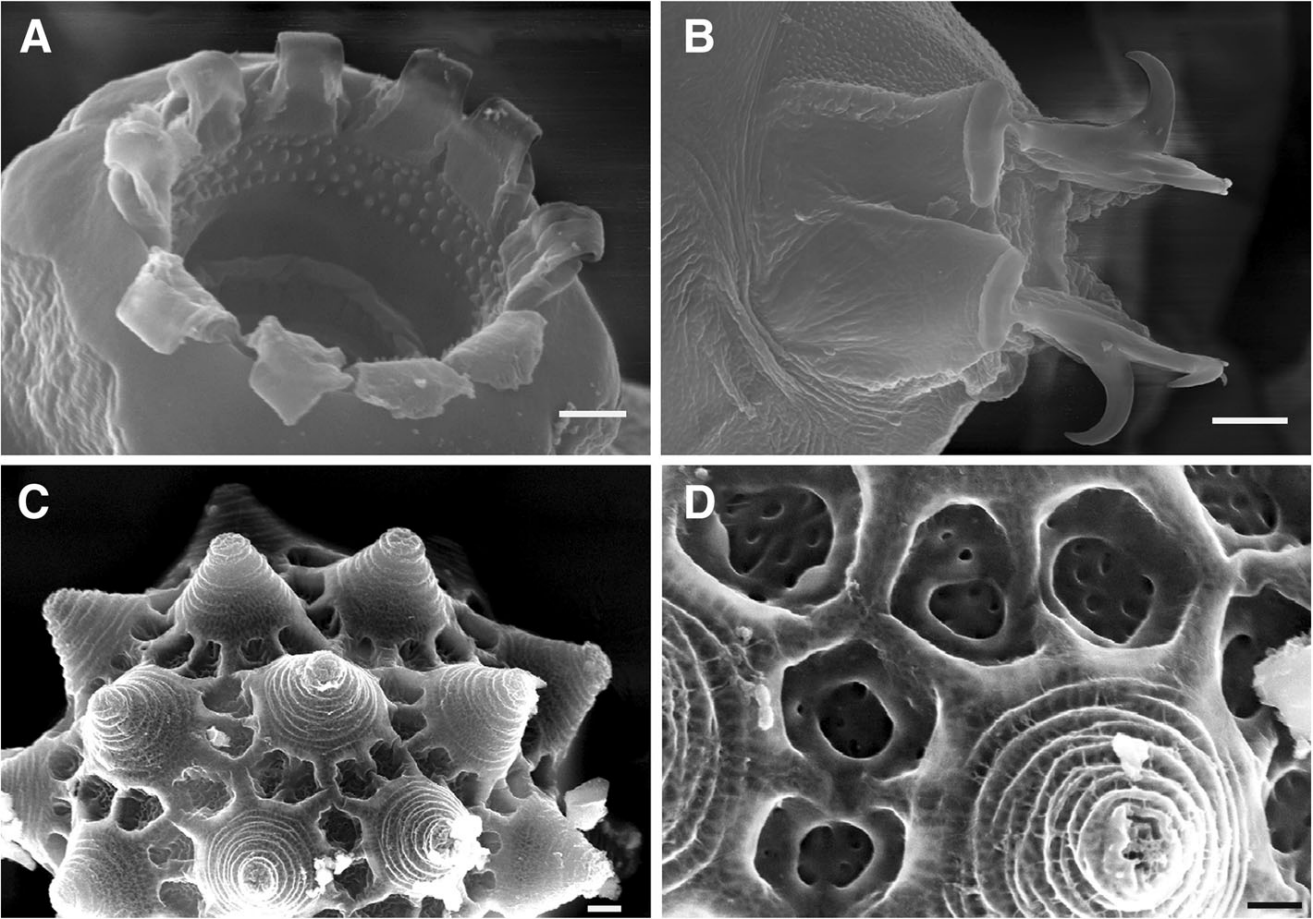
*Paramacrobiotus spatialis* sp. n., paratypes from Formigine (SEM). **a** Peribuccal lamellae and anterior band of teeth. **b** Claws of the fourth pair of legs. **c** Egg processes. **d** Detail of the areolae around the egg process. Bars: **a** and **d** = 2 μm, **b** and **c** = 5 μm

The eggs of the Italian populations have several similarities to those of the Clare Island (CI) population, but exhibit some differences too (see Discussion) (Figs. 5, 6 and 7). The egg processes can be always defined as truncated cones, but they can differ significantly among populations (Fig. 5). Under LM, the egg processes are reticulated in a similar way to those from the Irish population. The shell surface located around the bases of the processes always shows tiled structures (areolae), with holes and pits on their surface (Figs. 6, 7, 8, 9 and 10). In some cases, the qualitative characters of the eggs make it possible to distinguish between the Italian populations (see Discussion), although with some difficulty, due to the variability in shape and size of the egg shell morphology. When those qualitative characters were bound to clear molecular differences (*cox1*), they were able to be used to erect new species (see Discussion).

The morphometric characters related to the egg morphology (Table 2) show that the eggs of *P. richtersi* from Ireland (CI population) clearly differ from those of the Italian populations in several regards: the eggs from Ireland are larger than those from the Rocchetta, Passo Ballino, Piane di Mocogno, and Prodo populations; they have longer processes than those from Formigine, Gaggio, Passo Ballino and Pondel; they have wider processes than those originating from Rocchetta, Passo Ballino, and Prodo; and they have a lower number of processes per hemisphere than those from Rocchetta and Riccò. Moreover, quantitative differences are present among the Italian populations: the population of Gaggio has eggs larger than those of Rocchetta, Passo Ballino, Piane di Mocogno, and Prodo, and the population from Passo Ballino has eggs with the shortest processes with respect to all of the other Italian populations. The PCA analysis of the measures of the egg shell structures (Fig. 4c, d) shows only a defined separate cluster for the Clare Island eggs, while the other clusters are connected to each other.

### Reproductive biology

**Fig. 11:**
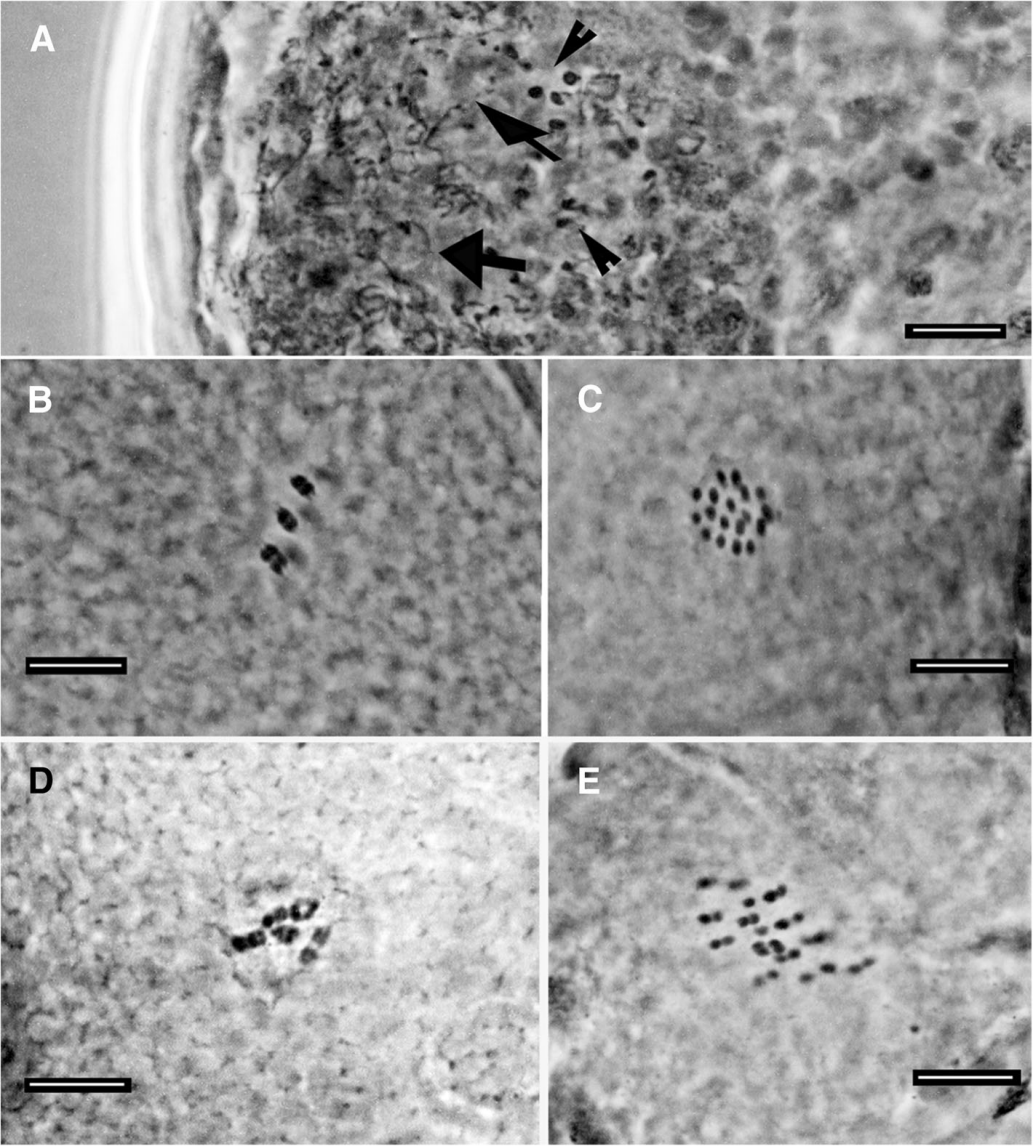
Male germ cells and chromosomes (orcein; PhC). **a** Testis with spermatids (arrowheads) and spermatozoa (arrows) of a male from Passo Ballino. **b** Bivalents in an oocyte of a female from Passo Ballino. **c** 18 univalents in an oocyte of a female from Rocchetta. **d** Bivalents in an oocyte of a female from Ospitaletto A. **e** Univalents in an oocyte of a female from Ospitaletto B. Bars = 10 μm

The Irish population from Clare Island (in both samples), and the Italian populations from Formigine, Gaggio, Passo Ballino, Piane di Mocogno, Prodo, Olbia, Andalo and Ospitaletto (OA) are bisexual as both males and females were found. With regard to the chromosome number, six bivalents were detected in the oocytes. The testis contains thin spermatozoa with a very long and thin helicoidal head (Figs. 3c, 9d) while in females, a seminal receptacle has never been observed. The Italian populations from Riccò, Rocchetta, Pondel and a second population from Ospitaletto (OB, collected about 1 km far from OA) are characterized by the absence of males and by the presence of 17–18 univalents in the oocytes of the females (Fig. 11), evidencing apomictic egg maturation.

### Molecular analyses

**Fig. 12:**
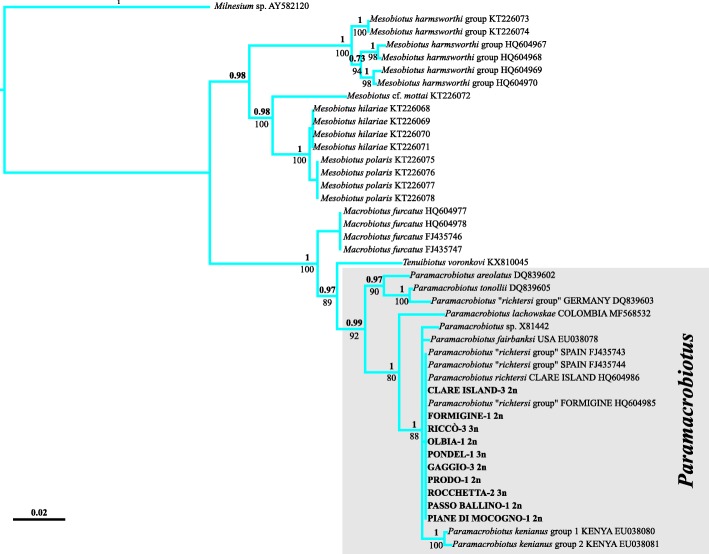
Tree resulting from the maximum likelihood (ML) and Bayesian inference (BI) analysis of 18S rRNA sequences in *Paramacrobiotus*. Values on branches indicate posterior probability values (above) and bootstrap values (below). The scale bar shows the number of substitutions per nucleotide position. GenBank accession numbers are reported after the taxon/population names

**Fig. 13:**
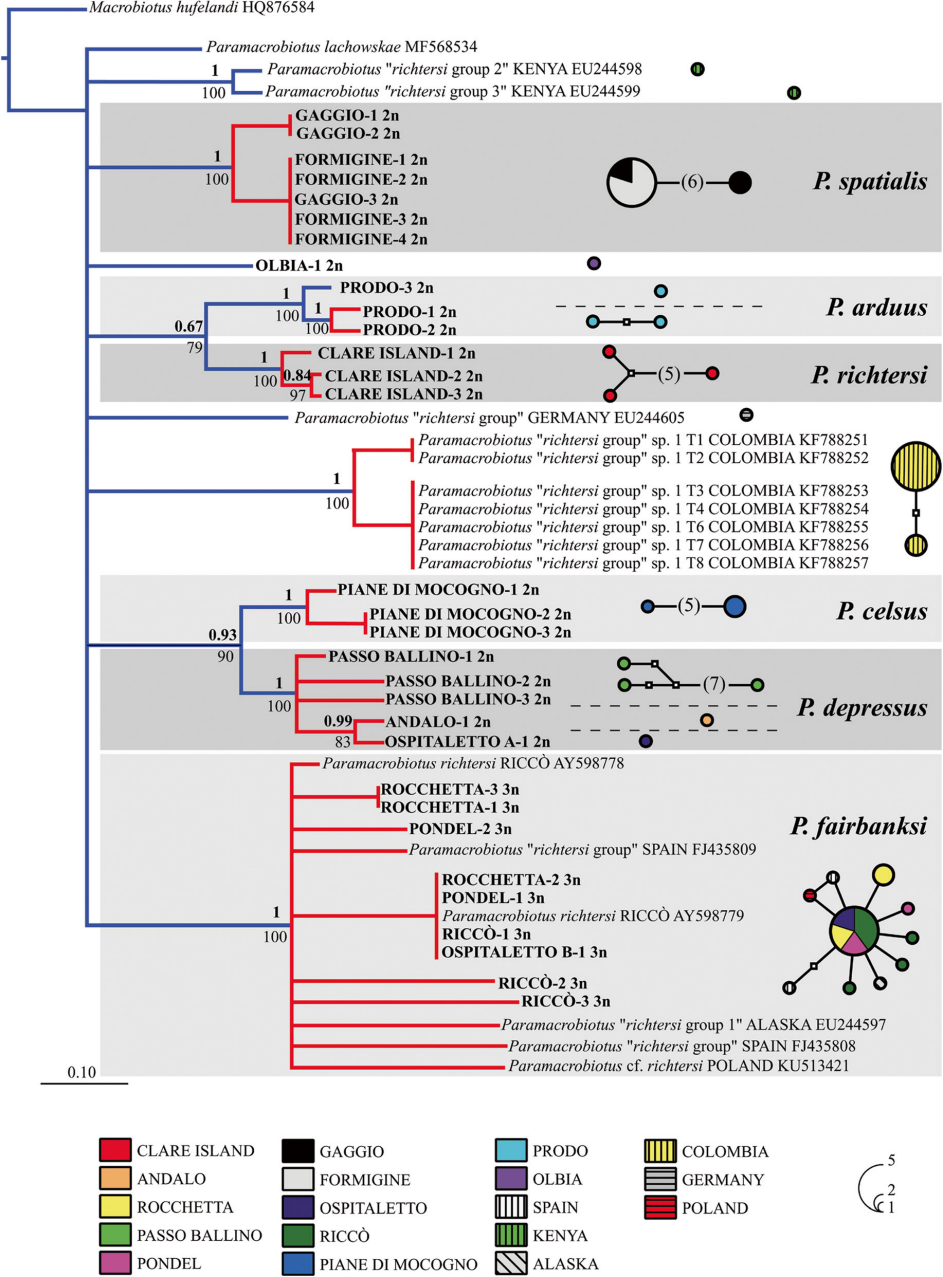
Tree resulting from the maximum likelihood (ML) and Bayesian inference (BI) analysis (left) and haplotype parsimony network (right) of cytochrome c oxidase subunit I (*cox1*) sequences in the *Paramacrobiotus richtersi* complex. Values on branches indicate posterior probability values (above) and bootstrap values (below). Results of the Poisson tree process (PTP) analysis are provided using differently coloured branches: putative species are indicated using transitions from blue to red coloured branches, the scale bar shows the number of substitutions per nucleotide position. Results of Network analysis are represented by networks: circles denote haplotypes, circle surface shows haplotype frequency, small white squares and number within parentheses indicate missing/ideal haplotypes, dotted lines demarcate networks falling below the value of the 95% connection limit. GenBank accession numbers are reported after the taxon/population names

The molecular analysis of the 18S gene (843 bp) revealed that all of the sequences obtained from the specimens of the different populations of *P. richtersi* analysed in this study had the same haplotype, shared with specimens from Madrid (Spain [56]). The phylogenetic analysis (18S, Fig. 12) showed the presence of two main clusters, one grouping *Paramacrobiotus tonollii* [57], *Paramacrobiotus areolatus* [58] and a specimen attributed to *P. richtersi* sampled in Germany, and the other clustering all remaining sequences of *Paramacrobiotus* comprising *Paramacrobiotus lachowskae* Stec et al., [59] (BI posterior probability value =1.0; ML bootstrap value =80%). On the other hand, the analysis of the mitochondrial *cox1* gene (606 bp) showed higher variability and provided new data on the genetic diversity of *P. richtersi* complex. Twenty-one haplotypes were found among the 31 analysed sequences (Fig. 13). Generally, no haplotypes were shared among any of the populations, with the exception of one haplotype found in four apomictic triploid populations (Pondel, Rocchetta, Ospitaletto B, and Riccò), and one haplotype found in two diploid populations (Gaggio and Formigine) (Fig. 13). Considering the data collected in this study and those present in GenBank, the mean genetic distance within each population is very low (always less than 1.5%). Among populations, the genetic distance was very low in regard to the apomictic triploid populations (0.1–0.3%) whereas a higher range of variability was exhibited when comparing the diploid populations among them (0.8–24.0%) (Table 3).

**Table 3:** Mean genetic distances (p-distance) computed among and within populations. All new haplotypes and those available in GenBank have been considered in the analysis, which was carried out on a 606 bp dataset. np, not possible

Population		Ploidy	Males	Within	Between															
					1	2	3	4	5	6	7	8	9	10	11	12	13	14	15	16
1	Formigine	2n	yes	0.000																
2	Riccò	3n	no	0.002	0.180															
3	Rocchetta	3n	no	0.001	0.183	0.002														
4	Gaggio	2n	yes	0.008	0.008	0.181	0.183													
5	Passo Ballino	2n	yes	0.014	0.194	0.181	0.178	0.192												
6	Piane di Mocogno	2n	yes	0.007	0.199	0.185	0.185	0.199	0.146											
7	Pondel	3n	no	0.002	0.182	0.002	0.002	0.182	0.179	0.184										
8	Olbia	2n	yes	np	0.185	0.195	0.195	0.191	0.180	0.206	0.197									
9	Prodo	2n	yes	0.014	0.212	0.214	0.217	0.213	0.209	0.207	0.215	0.186								
10	Clare Island (*P. richtersi*)	2n	yes	0.009	0.183	0.185	0.186	0.187	0.186	0.194	0.187	0.201	0.187							
11	Andalo	2n	yes	np	0.196	0.180	0.177	0.193	0.038	0.146	0.178	0.180	0.211	0.196						
12	Ospitaletto B	3n	no	np	0.178	0.001	0.001	0.178	0.179	0.185	0.001	0.196	0.216	0.185	0.178					
13	Ospitaletto A	2n	yes	np	0.200	0.189	0.185	0.198	0.047	0.149	0.186	0.178	0.207	0.186	0.050	0.186				
14	*P*. cf. *richtersi* (Madrid) FJ435808–9	?	no*	0.005	0.182	0.004	0.004	0.182	0.181	0.184	0.003	0.197	0.217	0.188	0.180	0.003	0.188			
15	*P. fairbanksi* EU244597	?	no*	np	0.183	0.003	0.003	0.183	0.181	0.185	0.002	0.198	0.218	0.189	0.180	0.002	0.188	0.004		
16	*P. kenianus* EU244598	?	no*	np	0.201	0.224	0.226	0.202	0.224	0.224	0.225	0.221	0.220	0.227	0.228	0.223	0.226	0.224	0.226	
17	*P. kenianus* EU244599	?	no*	np	0.205	0.224	0.224	0.204	0.229	0.230	0.224	0.215	0.232	0.230	0.236	0.223	0.231	0.223	0.224	0.041

*data from Schill et al. (2010) [34]

According to the PTP analysis (Fig. 13), eight different species were identified among the populations studied. These eight species corresponded to: i. *P. richtersi* (Clare Island population), mean genetic distance among specimens (MGD): 0.9%; ii. the populations from Formigine and Gaggio, MGD: 0.8%; iii. the population from Olbia; iv. the populations from Ospitaletto (OB), Pondel, Rocchetta, Riccò, Madrid (Spain), Kraków (Poland), and *P. fairbanksi*, MGD: 0.1–0.3%; v. two specimens from Prodo, MGD: 0.1%; vi. one specimen from Prodo; vii. the populations from Passo Ballino, Andalo, and Ospitaletto (OA), MGD: 3.8–5.0%; viii. the population from Piane di Mocogno, MGD: 0.7%. The MGD among putative species varied between 14.6 to 21.8% (Table 3, Additional file 2*:* Table S1). The parsimony network analysis (Fig. 13) confirmed these subdivisions, with the only exception being the breakup of the Andalo, Passo Ballino and Ospitaletto (OA) specimens into three different networks (3.8–5.3% p-distance).

## Discussion

### Species and cryptic diversity in the *Paramacrobiotus richtersi* group

*Paramacrobiotus richtersi* was originally described as *Macrobiotus richtersii* and collected in a moss from a salt marsh at Kinnacorra, Clare Island (Ireland) [21]. In Kinnacorra, we did not find a salt marsh, but in the same exact place some animals and related eggs of that species were found in a moss patch, as well as in turf, confirming its presence in the area reported in the original description.

Populations from the Italian localities examined in this study have animals that are very similar to *P. richtersi* from Ireland, with the same 18S sequence and the same peculiar shape of their spermatozoa (when males are present), but with very few differences in egg shells. An in-depth morphological analysis of the sclerified structures of the animals revealed no qualitative and quantitative differences among the specimens of the various Italian populations and *P. richtersi*. In some cases, a few differences were identified in the granulation of the leg cuticle (see Taxonomic account).

Differences among populations were found in the reproductive biology: meiotic (with bivalents) vs ameiotic (with univalents) oocyte maturation, diploid vs triploid chromosome number, presence vs absence of males in the population. When the population was unisexual (only females), it was also triploid and carried out ameiotic oocyte maturation. In contrast, when the population was bisexual (i.e. with females and males), it was diploid, with chromosome pairing during oocyte and spermatocyte maturation.

The egg shape is particularly helpful for recognizing the species belonging to Macrobiotidae [60], including *Paramacrobiotus* species. Kaczmarek et al. [27] classified *Paramacrobiotus* eggs into several typologies and provided a taxonomic key to identify the species of the genus, but despite the classification of egg morphotypes, difficulties in the species diagnosis remain. In the present study, the egg morphology of each population is similar, and characterized by intra-population (and not rarely within the same egg) variability. Nonetheless, some morphological and morphometric differences can be found among populations in the egg shape.

Molecular analyses based on 18S rRNA sequences revealed the presence of the same haplotype among all considered populations, whereas those based on *cox1* mtDNA sequences, together with the genetic distances among populations, revealed that the analysed sequences belong at least to eight different putative species. The number of species in the *richtersi* complex increases to 12 if all the *cox1* GenBank data are considered (four more species are represented by the two sequences of *P. kenianus*, and the sequences from Colombia and Germany; Fig. 13). These data are in contrast with the similarities in the morphologies of animals and eggs previously discussed. The genetic distances among the eight putative species can be very large (14.6-23.6%; Table 3), with values comparable to those found among genera in Eutardigrada (Additional file 3*:* Table S2).

The application of an integrative approach, which considered together the morphological differences in the eggs, the reproductive biology and the wide genetic distances among putative species, led to the description of four new species and two Unconfirmed Candidate Species (UCS) within the *P. richtersi* complex, and the re-description of two species (including the type species of the genus). The new species are *Paramacrobiotus depressus* sp. n. (Passo Ballino, Andalo, and Ospitaletto OA populations), *Paramacrobiotus spatialis* sp. n. (Formigine and Gaggio populations), *Paramacrobiotus celsus* sp. n. (Piane di Mocogno population) and *Paramacrobiotus arduus* sp. n. (Prodo population) (for species descriptions see Taxonomic account). The Italian populations of Riccò, Rocchetta, Pondel and Ospitaletto (OB) are attributed to *P. fairbanksi* according to the molecular analyses (genetic distance below 0.3%), and also because their animal and egg morphologies do not differ from the type specimens of this species.

In our view, the description of a new species (assigning to it a formal name) is warranted only when phenotypic differences are recognizable (e.g. in animal and egg morphologies, karyotypes, sex condition, or reproductive strategies). Based on this assumption, it was considered suitable to re-describe *P. fairbanksi* (see Taxonomic account), because in its original description it was differentiated from *P. richtersi* only on compensatory base changes in the secondary structure of the internal transcribed spacer 2 (ITS2) [34].

Evolutionary lineages identified only by molecular methods should be considered unconfirmed candidate species (UCS [61]). For this reason, the specimens from Olbia and the specimen of Prodo (PRODO-3) are considered cryptic species, or better, UCS, because the species delimitation methods separate them from *Paramacrobiotus spatialis* sp. n. and *Paramacrobiotus arduus* sp. n., respectively, but the animals and eggs are morphologically undistinguishable to those species. Following Padial et al. [61], these UCS should be defined with the combination of the binomial name of the most similar species (i.e. *P. spatialis* and *P. arduus*), followed (in square brackets) by the abbreviation “Ca” (for candidate) with an attached numerical code referring to the particular candidate species, and terminating with the author name and year of publication of the article (i.e. this paper) in which the lineage was first discovered. Therefore, the UCS from Olbia is defined as *Paramacrobiotus spatialis* [Ca1 Guidetti et al. 2019], and the UCS from Prodo is defined as *Paramacrobiotus arduus* [Ca1 Guidetti et al. 2019]. As has previously been stated: “The vouchers of the candidate species could be the GenBank accession numbers of the sequences used to propose the candidate status” [61], an alternative definition of these UCS are *Paramacrobiotus spatialis* [Ca1 MK041002] and *Paramacrobiotus arduus* [Ca1 MK041022].

There is not a universally accepted definition of cryptic species [1]. They can be viewed as discrete species that are difficult, or sometimes impossible, to distinguish morphologically. According to Bickford et al. [1], species difficult to distinguish can be placed into the category of pseudo-cryptic species once diagnosable characters are found. Our results suggest that the *P. richtersi* complex is formed by pseudo-cryptic species, as they do not show morphological differences among the animals, but are genetically distinct with differences in reproductive biology and/or a limited degree of differences in egg shell morphology (which in addition has a certain intraspecific variability). Therefore, only an integrated approach provides for a reasonable guarantee for their recognition.

Cryptic species have been already evidenced in several genera belonging to both classes of tardigrades (*Ramazzottius* [9, 17]; *Paramacrobiotus* [12]; *Richtersius* [13]; *Macrobiotus* [15]; *Echiniscus* [14]; *Echiniscoides* [10]), evidencing the surprisingly large magnitude of the phenomenon in the phylum.

### Geographic distribution

*Paramacrobiotus fairbanksi* has a wide distribution, being present in several Italian localities, in Spain [56], in Poland [62], and in Alaska [34]. The Polish specimen (KU513421) belongs to a parthenogenetic strain [62], it can be supposed that even the Alaskan, and Spanish populations are triploid and apomictic, as here verified for the four Italian ones. Nevertheless, *P. fairbanksi* is not the only thelytokous parthenogenetic species in *P. richtersi* complex; at least other two species have been found in Kenya (*Paramacrobiotus kenianus* Schill et al. [34]) and in Micronesia (*Paramacrobiotus palaui* Schill et al. [34]), even though their ploidy degree and their type of egg maturation remain unknown. In the present study, the amphimictic species seem to have a limited geographic distribution, in contrast with the wide distribution of *P. fairbanksi*. In a limited area of less than 400 km^2^ (corresponding to the polygon connecting the sampling sites in the Province of Modena, Italy) there are four species, three amphimictic and one apomictic; from this study, in Italy, at least five amphimictic species are present. Most of these have been found only in a single locality to date, as in the case of the true *P. richtersi*; *P. spatialis* sp. n. was found in two localities at a distance less than 16 km, and *P. depressus* sp. n. was found in three localities (two in the Alps, and one in the Po valley). The same phenomenon is observed in other genera of terrestrial tardigrades. Amphimictic species have often reduced/local distribution, while parthenogenetic species have widespread distribution (e.g. in *Echiniscus* and *Richtersius*; [8, 13, 63]). To date, the only exception is represented by the amphimictic species *Macrobiotus macrocalix* Bertolani and Rebecchi [60], identified with a molecular approach in Italy (in Apennines and Alps; [39]), Sweden [39], Poland [64], and Portugal [65], while the morpho-species was also cited in Poland [66], Austria [67], Albania [68], Spain [69], and Seychelles Islands [70]. The difference in distribution between apomictic and amphimictic populations can be explained by the difference in the potential of dispersal associated with the two types of reproduction. A parthenogenetic animal can colonize a new territory alone (Baker’s law; [71]), whereas a density-dependent reproduction is linked to amphimictic animals.

Three different thelytokous lineages are present in the *P. richtersi* complex. In general, the appearance of parthenogenetic lineages has been observed in several macrobiotids, isohypsibiids, hypsibiids and ramazzottiids, both in terrestrial and freshwater habitats [20, 72]. This indicates that this event is not rare in tardigrades, and confirms the idea that this situation can represent an advantage.

A further surprising phenomenon is that the diploid and triploid tardigrade species were never syntopic. In each examined sample only one species was identified. The same result was reported by Guidetti et al. [13] in a study on parthenogenetic and amphimictic species of the tardigrade genus *Richtersius,* while other studies on parthenogenetic species evidenced a mix of haplotypes within the same sample both for the *cox1* gene (in *Echiniscus* [8], in *Milnesium* [73]) and the ITS2 gene (but not for the *cox1* gene in *Ramazzottius* [74]). In many cases, the substrate colonized by the *P. richtersi* complex is very similar; therefore it is possible to speculate that the absence of syntopy could be due to the competition or mutual exclusion among species, such that only one species prevails. This hypothesis is supported by previous studies showing that in a very restricted area (i.e., the leaf litter surrounding a small group of hazelnut trees) in Formigine, a succession of species belonging to the *P. richtersi* complex was observed. For many years, only triploid female specimens were found [25, 75]. Then, a diploid amphimictic population completely substituted the triploid apomictic one [76]; this new population has been now identified as *P. spatialis* sp. n., but further evidences are needed to confirm this hypothesis of competition or mutual exclusion.

### *Paramacrobiotus* systematics

Kaczmarek et al. [27] proposed two new subgenera for *Paramacrobiotus*, whose names have been amended by Marley et al. [77] due to the assignment of the type species of the subgenera in contrast with the International Code of Zoological Nomenclature [78]. The actual subgenera are characterized by species with microplacoids in the pharynx for *Paramacrobiotus* (*Paramacrobiotus*), and species without microplacoids for *Paramacrobiotus* (*Amicrobiotus*). We consider the erection of these subgenera not well supported for two main reasons. The first is that they do not have molecular support, and the molecular data available are in contrast with their existence. Phylogenetic analyses carried out using 18S (this study, [12, 56]) evidenced that a specimen attributable to *P. richtersi,* or at least to the *P. richtersi* group (with microplacoid), belongs to the same lineage as *P. areolatus* and *P. tonollii* (both without microplacoid). Moreover, in our analysis, *P. lachowskae* (without microplacoid) clusters with species with microplacoid (i.e. all the new species here described, *P. richtersi*, *P. fairbanksi*, and *P. kenianus*; Fig. 12). The second reason is related to the fact that a microplacoid may be present or absent within several genera of eutardigrades (e.g. *Macrobiotus*, *Isohypsibius*, *Hypsibius*, *Diphascon*). The erection of new subgenera exclusively based on this character, without other molecular and/or morphological supports, can be risky especially because the evolutionary meaning of the microplacoid is not known. For these reasons, we do not think that the two subgenera are sufficiently supported, so they should be not considered valid.

Kaczmarek et al. [27] and Marley et al. [77] also proposed an emended diagnosis for the genus *Paramacrobiotus*, which we consider unnecessary because the new characters introduced in that diagnosis are either already used at superfamily level, or not shared by all species, or, lastly, not useful for differentiating genera or subgenera (e.g. relative length of the macroplacoids or shape of the lunules).

## Conclusions

A group of pseudo-cryptic species (*P. richtersi* complex) has been identified within the tardigrade genus *Paramacrobiotus*. Within this group, only a combined integrative approach, which considered the morphological differences in the eggs, the reproductive biology and the wide genetic distances among putative species, led to the identification of differences among phylogenetic lineages. This has led to the description of four new species and two UCS, other than to the re-description of both the type species of the genus (*P. richtersi*) and the unique parthenogenetic, triploid species of the complex (*P. fairbanksi*). All other species in the complex were amphimictic, diploid species; the diploid and triploid species were never syntopic. The parthenogenetic species showed a very wide distribution, being present in different continents, while the amphimictic species showed a very limited or punctiform distribution. The difference in distribution between apomictic and amphimictic populations can be explained by the difference in the potential of dispersal associated with the two types of reproduction.

## Taxonomic account

Redescriptions of *Paramacrobiotus richtersi* and *Paramacrobiotus fairbanksi*; descriptions of *Paramacrobiotus depressus* sp. n., *Paramacrobiotus spatialis* sp. n., *Paramacrobiotus celsus* sp. n. and *Paramacrobiotus arduus* sp. n.

### *Paramacrobiotus richtersi* (Murray, 1911) (re-description)

*Material examined*: neotype (slide C2714–14 in the Bertolani collection at the Department of Life Sciences, University of Modena and Reggio Emilia) and 15 specimens from the type locality (Clear Island, Ireland).

### Description

*Neotype*. Specimen 487.6 μm in length (morphometric data in Table 2, Additional file 1: Table S3). Male. Without eyes. Lactescent. Cuticle smooth, without pores. Fine granulation of small non-uniform granules (max diameter 0.5–0.6 μm) on the distal and lateral surface of the first three pairs of legs and, more visible, on the medial and posterior part on the hind legs (Fig. 2e, f). Mouth surrounded by large and square-shaped peribuccal lamellae. Buccal armature in the oral cavity: anterior band at the beginning of the buccal ring (at the base of the peribuccal lamellae) with 3–4 rows of many small round teeth of different size; posterior band at the beginning of the buccal tube with a crown of triangular or bicuspid strong teeth; transverse crests: dorsally three long crests, ventrally two very short lateral crests and a line of three round teeth of similar size instead of the median crest (Fig. 2c, d). Buccal tube 43.7 μm in length and 9.1 μm in internal width, with an evident ventral lamina. Stylet supports inserted at 35.2 μm from the beginning of the buccal tube (*pt* 80.5). Stylet furca well developed. Slightly oval pharyngeal bulb containing large triangular apophyses, followed by three rod-shaped macroplacoids, the third with an evident caudal constriction, and an evident microplacoid (Fig. 2b). Macroplacoid row length 26.1 μm (*pt* 59.7), first macroplacoid 9.5 μm in length (*pt* 21.7), second 7.8 μm (*pt* 17.9), third 9.0 μm (*pt* 20.6). Between the third macroplacoid and the microplacoid a thin sclerified line present. Microplacoid 4.6 μm in length, similar to a grape-seed (apex backwards), not in line with the curvature of the macroplacoids but parallel to the axis of the buccal tube and positioned at a long distance (longer than the microplacoid length) from the third macroplacoid. Claws of *hufelandi* type, with a small triangular basal tract without internal septum defining a distal part, and a very thin base (Fig. 2e, f). Evident accessory points in the main claw branches. Small smooth lunules in the first three pairs of legs, larger in the hind legs. External claw on the third pair of legs, measured including the evident accessory points, 14.5 μm in length (*pt* 33.2); posterior claw on the fourth pair of legs 13.3 μm in length (*pt* 30.5). Weak transverse bar under the two claws but at a distance from them in the first three pairs of legs (Fig. 2e).

*Other material from the same sample*: Animals from 398 μm to 617 μm in length (for other morphometric data see Table 2). In lateral view, s-shaped (with a double curvature; Fig. 2c) and large buccal tube. Buccal armature in the oral cavity as in the neotype; sometime a small, supernumerary lateral round tooth present beside each lateral ventral crests, or ventral crests interspersed by a single small crest or a line of two or three round teeth of similar size.

Bisexual, with males and females (in both Clare Island samples; Table 1). Six bivalents in the equatorial plane in the oocytes (Fig. 3); spermatids and spermatozoa observed within the testis (with LM, after orcein staining; Fig. 3) and even on an egg shell surface (SEM image; Fig. 8d). When ripe, the head of the spermatozoon looks thread-like (filiform), particularly long and helicoidal shaped.

Eggs (Figs. 5, 6, 9, Additional file 4: Figure S1) ornamented, 70–75 μm in diameter excluding processes, and laid freely. Egg processes high and very often as inverted funnels ending with a very short and wide tube, in few cases as elongated truncated cones, or cones. Distal part of the processes flat or rounded at the end, slightly corrugated with SEM. Processes 12–18 μm high with an inner diameter at their base of 17–21 μm (Table 2). Number of processes per hemisphere varying from 13 to 17 (Table 2). With LM, process wall reticulated (due to inner trabecular structures), with meshes increasing in size with the length of the process and passing from the base to the top. With SEM, surface of the processes often with concentric circles. Egg shell among the processes tiled (areolate), with 9–11 hollow tiles around each process, not in contact with one another but separated by fine meshes (with LM). The inner part of the tiles sculptured with small round pits of different size and not uniformly distributed.

*Type repositories*: The neotype (slide C2714-S14), 13 specimens and nine eggs of the same population are mounted on slides in Faure Berlese mounting medium and deposited in the Bertolani collection at the Department of Life Sciences, University of Modena and Reggio Emilia, Italy, two specimens are deposited in the Museum of Natural History of Verona, Italy.

*Differential diagnosis*: *P. richtersi* has macroplacoids and no eye spots, so it differs from all the *Paramacrobiotus* species without microplacoid and/or with eye spots, and it differs from the species with microplacoid and without eye spots for the following characters: *-Paramacrobiotus alekseevi* [79]: Animals: for smaller latero-ventral transversal crests without denticulate anterior margins in the buccal armature, absence of small teeth in the hind lunules and presence of a fine granulation on first three pairs of legs. Eggs: for more regular, cone-shaped egg processes and for the lower number of tiles around each process (10–12 in *P*. *alekseevi*). *-Paramacrobiotus chieregoi* [80]: Eggs: for shorter egg processes (about 28 μm in height in *P*. *chieregoi*) of more regular conical shape, and for the presence of tiles around the process base. *-Paramacrobiotus danielisae* [81]: Animals: for the absence of fine sculpture of very small polygons on the cuticle surface. *- Paramacrobiotus halei* [82]: Animals: for the absence of very small tubercles on the cuticle surface, and for the presence of the median ventral crest subdivided in smaller pieces in the buccal armature. Eggs: for longer processes. *-Paramacrobiotus garynahi* [83]: Animals: for the absence of oval pores on the cuticle surface, and for median ventral crest subdivided in smaller pieces in the buccal armature. Eggs: for shorter egg processes. *-Paramacrobiotus gerlachae* [84]: Animals: for the presence of a fine granulation on the first three pairs of legs and median ventral crest subdivided in smaller pieces in the buccal armature. Eggs: for longer processes and the shape of the processes, as long cones. *-Paramacrobiotus hapukuensis* [85]: Animal: for the presence of granulations in all pairs of legs, and for the median ventral crest subdivided in smaller pieces in the buccal armature. Eggs: for the shape of processes without a finger-shaped terminal portion. *-Paramacrobiotus lorenae* [86]: Eggs: for the shape of processes without a finger-shaped terminal portion, and an evident reticulated surface (with LM). *-Paramacrobiotus peteri* [87] Animal: for the presence of granulations in all pairs of legs, and for the median ventral crest subdivided in smaller pieces in the buccal armature. Eggs: for the larger processes, without apices subdivided into a number of points, and for the larger number of areolae around each process (6–7 in *P. peteri*).

### *Paramacrobiotus fairbanksi* Schill, Förster, Dandekar and Wolf, 2010 (redescription)

*Material examined*: Seven paratypes and 10 eggs (from a culture of the type population by R. Schill) and animals and eggs from the Riccò, Rocchetta and Pondel populations all deposited in the Bertolani collection at the Department of Life Sciences, University of Modena and Reggio Emilia.

### Description

Animal length from 254 μm to 791 μm (morphometric data in Table 2, Additional file 1: Table S3). No eye spots. Lactescent. Cuticle smooth, without pores. Fine granulation on a small cuticular surface lateral to the external claws on the first three pairs of legs and larger in the medial-basal ventral part of the hind legs, similar in shape and size to that of *P. richtersi* (Additional file 5*:* Figure S2). Square peribuccal lamellae around the mouth opening. Buccal armature (Additional file 5: Figure S2C, D) with an anterior wide band of fine teeth at the level of the base of peribuccal lamellae, an evident posterior row of long triangular or bicuspid teeth at the beginning of the buccal tube, and transversal crests. Transverse crests: dorsally, three long crests, and ventrally two lateral shorter crests, sometime fragmented, and one median crest or two-three teeth. Buccal tube s-shaped and large, with evident ventral lamina. Stylet support insertion at 75–83% of the buccal tube length. Stylet furca well developed. Within an oval pharyngeal bulb, large triangular apophyses and three rod-shaped macroplacoids, and one large and comma-shaped microplacoid present (Additional file 5: Figure S2B). Third macroplacoid with a clearly visible constriction near to its caudal end. Thin sclerified line, longer than the macroplacoid length, connecting the third macroplacoid to the microplacoid. Shape and position of the microplacoid as in *P. richtersi*. Claws of *hufelandi* type, with a small triangular basal tract without internal septum defining a distal part, and a very thin base (Additional file 5: Figure S2E, F). Evident accessory points in the main claw branches. Small smooth lunules, much larger on the hind legs. One weak transverse bar often with double curvature on the first three pairs of legs, at a distance from the lunules.

Without males. All the Italian populations here considered with 17–18 univalents in the oocytes (Fig. 11c).

Eggs from the type locality ornamented, 62–83 μm in diameter without processes, and laid freely. Egg processes 11–15 μm high and with an inner diameter at their bases of 11–21 μm. Processes as truncated cones with jagged relieved apex and reticulated surface (with LM). Reticulation with small meshes of various size, the size not in relationship with the mesh position on the process. From 17 to 22 processes per hemisphere. Single or often double (in this case, 5–6) large areolae (tiles) around the processes. Within areolae, pits evidencing a cribrose surface, similar to that in *P. richtersi*.

Eggs from Riccò (Figs. 5, 6, Additional file 6: Figure S3, Additional file 7: Figure S4): 18–22 processes per hemisphere. Processes as truncated cones with a jagged apex; apex in some cases narrow. Five tiles, each subdivided in two, around each process, with pits on the bottom (cribrose surface; Additional file 7: Figure S4D). Eggs from Rocchetta (Figs. 5, 6, 7 and 8f): processes as truncated cones with a jagged apex, 19–25 processes per hemisphere. About six large areolae around each process, often subdivided in two by a more or less thin crest. Eggs from Pondel (Figs. 5, 6, 7 and 8c): 16–25 processes per hemisphere. Surface of the processes with relieved reticulation. Apex of the processes jagged. Large areolae (5–7) around processes, each one sometimes subdivided in two by a crest. Edges of the areolae relatively thick. Surface of areolae cribrose (with SEM), with white dots (with LM, PhC).

*Differential diagnosis*: *P. fairbanksi* differs from the other species described or re-described in this paper by the presence of triploidy and apomictic parthenogenesis and, as consequence, of thelytoky. To date, only molecular data distinguish this species from *P. kenianus* and from *P. palaui*, both ascertained as parthenogenetic.

*Paramacrobiotus fairbanksi* has macroplacoids and no eye spots, so it differs from all the *Paramacrobiotus* species without microplacoid and/or with eye spots, and it differs from the species with microplacoid and without eye spots for the following characters: *-P. alekseevi*: Animals: for smaller latero-ventral transversal crests without denticulate anterior margins in the buccal armature, and absence of small teeth in the hind lunules and presence of a fine granulation on first three pairs of legs. Eggs: for more regular, cone-shaped, egg processes without cap-like vesicular structures on their apices and for the presence of tiles with a central septum (double tiles) around each process. *-P. chieregoi*: Eggs: for shorter egg processes (about 28 μm in height in *P*. *chieregoi*) of more regular conical shape, and for the presence of tiles around the process base. *-P. danielisae*: Animals: for the absence of fine sculpture of very small polygons on the cuticle surface. Eggs: for shorter processes. *- P. halei*: Animals: for the absence of very small tubercles on the cuticle surface. Eggs: for longer processes. *-P. garynahi*: Animals: for the absence of oval pores on the cuticle surface, and median ventral crest subdivided in small pieces in the buccal armature. Eggs: for shorter egg processes without cap-like structures at their apices. *-P. gerlachae*: Animals: for the median ventral crest generally subdivided in small pieces in the buccal armature, and for the presence of a granulation in the first three pairs of legs. Eggs: for the shape of the processes, as truncated cones with jagged relieved apex. *-P. hapukuensis*: Animal: for the presence of granulations in all pairs of legs. Eggs: for the shape of processes without a finger-shaped terminal portion. *-P. lorenae*: Eggs: for the shape of processes without a finger-shaped terminal portion, and an evident reticulated surface (with LM). *-P. peteri*: Animal: for the presence of granulations in all pairs of legs. Eggs: for the larger processes, without apices subdivided into a number of points, and for the larger number of areolae around each process (6–7 in *P. peteri*).

### *Paramacrobiotus spatialis* sp. n. (Figs. 5, 7, 8, 10, Additional file 8: Figure S5, Additional file 9: Figure S6)

ZooBank registration: urn:lsid:zoobank.org:act:8D1168E4-CE25-43D1-84C3-B6C6A16378CD.

*Holotype.* slide C2680-20a. Formigine (Modena), ITALY; 44°N 34.253, 010°E 50.892.

*Paratypes*. Same data as for holotype.

*Other material*: sample C2698. Gaggio (Villa Sorra), Modena, Italy; 44°N 37.736, 011°E 02.234.

*Etymology*: *spatialis* (latin) means spatial, due to the fact that this species has flowed in the space in 2007 (LIFE-TARSE Mission on FOTON-M3) [88].

### Description

*Holotype*. Animal length 395.0 μm (morphometric data in Table 2, Additional file 1: Table S3). Without eyes. Lactescent. Sex undetermined. Cuticle smooth, without pores. A granulation on the cuticle of the hind legs present, similar to that of *P. richtersi*, but with more spaced and smaller granules (diameter 0.3–0.4 μm). Slight granulation in the first three pairs of legs present. Mouth surrounded by long tape-shaped peribuccal lamellae. Buccal armature with an evident anterior band of 3–4 rows of small teeth positioned at the base of the buccal lamellae, a posterior row of very large cusped teeth, sometimes joint two by two, in the caudal part of the mouth (beginning of the buccal tube) followed by transversal crests (Additional file 8: Figure S5C, D). Transversal crests: dorsally, three long crests; ventrally, two short lateral crests and a median crest subdivided in three round teeth in line, the central tooth the shortest. Buccal tube length 45.4 μm, internal width 7.6 μm. Insertion of the stylet supports at 34.9 μm (*pt* 76.9). In the pharyngeal bulb, large apophyses and three rod-shaped macroplacoids, the third with an evident terminal constriction (Additional file 8: Figure S5b). Macroplacoid row length 26.5 μm (*pt* 58.4), first macroplacoid 9.0 μm in length (*pt* 19.8), second macroplacoid 7.0 μm in length (*pt* 15.4), third macroplacoid 9.9 μm in length (*pt* 21.8). Microplacoid grape seed-shaped, big (4.1 μm in length), far from the macroplacoids (more than its length) and not in line with them, but positioned parallel to the buccal tube axis. Thin sclerified line connecting the third macroplacoid to the microplacoid. Claws of *hufelandi* type, their bases with smooth lunules larger in the hind legs (Additional file 8: Figure S5e, f). External claw in the third pair of legs 13.4 μm in length (*pt* 29.5); posterior claw of the hind legs 14.2 μm in length (*pt* 31.3). Bar under the claws of the first three pairs of legs not evident.

The other animals of the population were similar in appearance to the holotype. Males and females present (namely: 57 males, 62 females and 43 undifferentiated).

Quantitative data for the species are presented in Table 2.

Eggs ornamented (Figs. 5, 7, 10, Additional file 9: Figure S6), 65–75 μm in diameter without processes, and laid free. From 15 to 23 processes per hemisphere. Processes 13–16 μm high, with variable profile (Fig. 5), generally with large base and a narrow distal part, often not really flat but ending with concentric circles of spiniform tubercles or irregular crests. Processes reticulated (with LM) and sometime scaled. Generally five areolae (tiles) around the processes, each tile with a crest inside forming two hollows per tile (resembling 10 areolae around each process), tiles not so wide as in *P. fairbanksi*. Surface of the hollows with pits much more concentrated in the central part.

The animals and the eggs from Gaggio have been attributed to the same species because they are very similar to those of Formigine, including the presence of males and females and the presence of spiniform tubercles on the top of the egg processes. Egg diameter without processes varies from 68 to 78 μm; the process height from 14 to 17 μm. The number of processes per hemisphere varies from 18 to 26.

*Type locality*: Formigine (Modena), Italy (sample C2680; 44°N 34.253, 010°E 50.892). Other locality: Gaggio (Villa Sorra), Modena, Italy (sample C2698).

*Type repositories*: the holotype (slide C2680-S20a), 277 paratypes and 22 eggs, together with, 11 specimens and nine eggs from Gaggio, have been mounted on slides in Faure-Berlese fluid and deposited in the Bertolani collection at the Department of Life Sciences, University of Modena and Reggio Emilia, Italy, three paratypes are deposited in the Museum of Natural History of Verona, Italy.

*Differential diagnosis*: *Paramacrobiotus spatialis* sp. n. is similar to *P. richtersi* and *P. fairbanksi.* It differs from *P. richtersi* in some characters of the eggs and for a less evident granulation on the legs. Eggs processes shorter, not funnel-like but as truncated cones and with an irregular distal part; tiles larger and more homogeneous. It differs from *P. fairbanksi* for the presence of males, a diploid number of chromosomes with pairing in the egg, and sperm maturation, a less evident granulation on the legs and for narrower areolae on the egg shell.

*Paramacrobiotus spatialis* sp. n. has macroplacoids and no eye spots, so it differs from all the *Paramacrobiotus* species without microplacoid and/or with eye spots, and it differs from the species with microplacoid and without eye spots for the following characters: *-P. alekseevi*: Animals: for smaller latero-ventral transversal crests without denticulate anterior margins in the buccal armature, absence of small teeth in the hind lunules and presence of a fine granulation on first three pairs of legs. Eggs: for more regular, cone-shaped, egg processes without cap-like vesicular structures on their apices, and the lower number of tiles around each process (10–12 in P. *alekseevi*). *-P. chieregoi*: Eggs: for shorter egg processes (about 28 μm in height in *P*. *chieregoi*) of more regular conical shape, and for the presence of tiles around the process base. *-P. danielisae*: Animals: for the absence of fine sculpture of very small polygons on the cuticle surface. Eggs: for shorther processes. *-P. garynahi*: Animals: for the absence of oval pores on the cuticle surface, and median ventral crest subdivided in smaller pieces in the buccal armature. Eggs: for shorter egg processes without cap-like structures at their apices. *-P. gerlachae*: Animals: median ventral crest subdivided in smaller pieces in the buccal armature, and for the presence of a granulation in the first three pairs of legs. Eggs: for surface of the areolae around processes with evident pits (areolae are thickened and with a faint sculpture of some circular pores in *P. gerlachae*). *- P. halei*: Animals: for the absence of very small tubercles on the cuticle surface. Eggs: for longer processes. *-P. hapukuensis*: Animal: for the presence of granulations in all pairs of legs. Eggs: for the shape of processes without a finger-shaped terminal portion. *-P. lorenae*: Eggs: for the shape of processes without a finger-shaped terminal portion, and an evident reticulated surface (with LM). *-P. peteri*: Animal: for the presence of granulations in all pairs of legs. Eggs: for the larger processes, without apices subdivided into a number of points, and for the larger number of areolae around each process (6–7 in *P. peteri*).

*Remarks*: In the sample (C2702 from Olbia) a second species, *Paramacrobiotus spatialis* [Ca1 MK041002], was identified by molecular analyses, but resulted morphologically indistinguishable from *Paramacrobiotus spatialis* sp. n.

Eggs of *P. spatialis* [Ca1 MK041002] (Figs. 5, 7, Additional file 10: Figure S7) with 15–20 processes per hemisphere. Truncated cone-shaped processes, with irregular or slightly prolonged apex, not jagged. Reticulation almost regular. Tiles relatively small, about 6 in number, some of them subdivided in two parts.

Thirty-two specimens and 13 eggs mounted in Faure-Berlese fluid of this cryptic species are deposited in the Bertolani collection at the Department of Life Sciences, University of Modena and Reggio Emilia, Italy.

### *Paramacrobiotus depressus* sp. n. (Figs. 5, 7, 8, 11, Additional file 11: Figure S8, Additional file 12: Figure S9 and Additional file 13: Figure S10)

ZooBank registration: urn:lsid:zoobank.org:act:3F0E9DCF-8135-4901-A2D1-EAE0635D7093.

*Holotype*. slide C2693–7. Passo Ballino (Trento), Italy; 45°N 58.724, 010°E 49.374.

*Paratypes*. Same data as for holotype.

*Other material*: sample C2762, Andalo (Trento), Italy; 46°N 09.742, 010°E 59.455. Sample C2794, Ospitaletto (Ospitaletto A, OA), (Modena) Italy; 44°N 26.521, 010°E 53.207.

*Etymology*. from latin *depressus*, of low level, referred to the egg processes.

#### Description

*Holotype*. Animal length 651.7 μm (morphometric data in Table 2, Additional file 1: Table S3). Without eyes. Lactescent. Sex undetermined. Cuticle smooth, without pores. Weak granulation (granules diameter 0.3–0.4 μm) on the hind legs, almost absent in the first three pairs of legs. Mouth surrounded by peribuccal lamellae. Buccal armature: anterior band formed by a narrow band of fine teeth at the beginning of the buccal ring (at the base of the buccal lamellae); posterior band formed by one row of evident teeth at the beginning of the buccal tube (teeth as long triangles with the apex toward the mouth opening in the dorsal side, more roundish in the ventral side); transverse crests: dorsally, three long and rectangular crests, ventrally, two long and comma-shaped lateral crests and one short and roundish median crest (Additional file 11: Figure S8C). Buccal tube, 49.5 μm in length and 8.4 μm (*pt* 17.0) in internal width, slightly turned at the level of the stylet support insertion. Insertion of the stylet supports at 81.7% of the buccal tube length. Pharyngeal bulb with pharyngeal apophyses, three rod-shaped macroplacoids and a big microplacoid similar to a half grape seed with its apex caudal (Additional file 11: Figure S8B). Placoid row length 32.1 μm (*pt* 64.9); first, second and third macroplacoid 10.4 μm (*pt* 21.0), 9.3 μm (*pt* 18.7) and 11.3 μm (*pt* 22.8) in length, respectively. Third macroplacoid with a slightly visible constriction near to its caudal end. Microplacoid far from the third macroplacoid (more than the microplacoid length) and parallel to the axis of the buccal tube. Thin sclerified line connecting the third macroplacoid to the microplacoid. Claws of *hufelandi* type, with small and smooth lunules, a little larger in the fourth pair of legs (Additional file 11: Figure S8D, F). External claw of the third pair of legs 12.6 μm in length (*pt* 25.5), posterior claw of the fourth pair of legs 12.6 μm in length (*pt* 25.4). Small bar made by black granules (with PhC) under the internal claws of the first three pairs of legs.

*Other material from the same sample*: paratypes can differ from the holotype in the shape of the median ventral crest (formed by two or three teeth instead of one crest) and in a better evidence of a granulation on the external surface of all legs. Paratype morphometric data are reported in Table 2.

Males and females present. Six bivalents in the oocytes (Fig. 11b).

Eggs ornamented, 56.2–66.2 μm in diameter excluding processes, and laid free (Figs. 5, 7, 8b Additional file 12: Figure S9, Additional file 13: Figure S10). Egg processes, 16–23 per hemisphere, in shape of relatively short cones (9.3–12.4 in height), only in some eggs, processes with short cylindrical and relatively large extremities. Process surfaces scaled but not evidently and with a thin reticulation with tight meshes. Areolae around the process base with wide tiles, often 5–6 in number but subdivided in two parts by an internal crest. Surface of areolation with pits forming a cribrose area.

Eggs from Ospitaletto (OA): processes as truncated cones, with a jagged apex, 20–24 processes per hemisphere. Areolae around the processes often nine.

No valuable eggs have been found in Andalo.

*Type locality*: Passo Ballino (Trento), Italy (sample C2693, 45°N 58.724, 010°E 49.374). Other localities: Andalo (Trento), Italy (C2762); Ospitaletto (Ospitaletto A, OA), (Modena), Italy (C2794).

*Type repositories*: the holotype (slide C2693-S7), 8 paratypes and 10 eggs are mounted on slides in Faure Berlese mounting medium and deposited in the Bertolani collection at the Department of Life Sciences, University of Modena and Reggio Emilia, Italy, 2 paratypes are deposited in the Museum of Natural History of Verona, Italy.

*Differential diagnosis:* This species clearly differs from *P. richtersi* for the different shape of the egg processes, which are not funnel-like, and for a weaker granulation on the legs. It differs from *P. fairbanksi* in egg shape (mainly for shorter processes), in sex condition (males absent in *P. fairbanksi*) and also for a weaker granulation on the legs. It differs from *P. spatialis* sp. n. in having a smaller egg diameter, shorter processes (high so as large at their bases only in *P. depressus* sp. n.) and shorter areolae.

*Paramacrobiotus depressus* sp. n. has macroplacoids and no eye spots, so it differs from all the *Paramacrobiotus* species without microplacoid and/or with eye spots, and it differs from the species with microplacoid and without eye spots for the following characters: *-P. alekseevi*: Animals: for smaller latero-ventral transversal crests without denticulate anterior margins, for the ventral median crest not subdivided in smaller pieces in the buccal armature, and absence of small teeth in the hind lunules. Eggs: for more regular, cone-shaped, egg processes without cap-like vesicular structures on their apices. *-P. chieregoi*: Animals: ventral median crest not subdivided in smaller pieces in the buccal armature. Eggs: for shorter egg processes (about 28 μm in height in *P*. *chieregoi*) of more regular conical shape, and for the presence of tiles around the process base. *-P. danielisae*: Animals: for the absence of fine sculpture of very small polygons on the cuticle surface. Eggs: for shorter processes. *-P. garynahi*: Animals: for the absence of oval pores on the cuticle surface, and median ventral crest subdivided in smaller pieces in the buccal armature. Eggs: for shorter egg processes without cap-like structures at their apices. *-P. gerlachae*: Eggs: for shorter egg processes, and for areolae around the process base with wide tiles, often 5–6 in number but subdivided in two parts by an internal crest. *-P. halei*: Animals: for the absence of very small tubercles on the cuticle surface. *-P. hapukuensis*: Animal: for the presence of granulations in all pairs of legs. Eggs: for the shape of processes without a finger-shaped terminal portion. *-P. lorenae*: Eggs: for the shape of processes without a finger-shaped terminal portion, and an evident reticulated surface (with LM). *-P. peteri*: Animal: for the presence of granulations in all pairs of legs. Eggs: for the larger processes, without apices subdivided into a number of points, and for the larger number of areolae around each process (6–7 in *P. peteri*).

### *Paramacrobiotus celsus* sp. n. (Figs. 5, 6, Additional file 14: Figure S11, Additional file 15: Figure S12)

ZooBank registration: urn:lsid:zoobank.org:act:3A36FEAB-A395-4D1B-9FB6-A48247D40F48.

*Holotype*. slide C2112–10. Piane di Mocogno (Modena), Italy; 44°N 16.775, 010°E 40.133.

*Paratypes*. Same data as for holotype.

*Etymology*. *celsus* (latin), high, referred to the egg processes.

#### Description

*Holotype*. Animal length 527.1 μm (morphometric data in Table 2, Additional file 1: Table S3). Without eyes. Lactescent. Sex undetermined. Cuticle smooth, without pores. A granulation on the surface close to the posterior claw of the hind legs, with granules similar in size to those of *P. richtersi*, but not so evident. The granulation is visible in the first three pairs of legs. Mouth surrounded by wide square peribuccal lamellae. Buccal armature (Additional file 14: Figure S11C, D): anterior band at the beginning of the buccal ring (at the base of the buccal lamellae) with a band of small teeth, posterior band at the beginning of the buccal tube (caudal part of the mouth) with a row of large cusped teeth, sometimes jointed two by two; dorsally followed by three dorsal and ventral transversal crests. Ventral crests shorter and with a triangular median crest. Buccal tube length 53.3 μm, internal width 8.2 μm (*pt* 15.4). Insertion of the stylet supports at 42.1 μm (*pt* 79.1). Pharyngeal bulb with large apophyses, three rod-shaped macroplacoids (the third with an evident terminal constriction) and a microplacoid (Additional file 14: Figure S11B). Macroplacoid row length 35.5 μm (*pt* 66.6), first macroplacoid 11.2 μm (*pt* 21.1), second macroplacoid 9.0 μm (*pt* 16.9), third macroplacoid 11.6 μm (*pt* 21.8) in length. Big and drop-shaped microplacoid (4.7 μm in length), far from the macroplacoids and not in line with them, but positioned parallel to the buccal tube. Thin sclerified line connecting the third macroplacoid to the microplacoid. Claws of *hufelandi* type, with smooth lunules at their base, larger in the hind legs (Additional file 14: Figure S11E–G). External claw in the third pair of legs 15.1 μm in length (*pt* 28.4); posterior claw of the hind legs 16.4 μm in length (*pt* 30.8). A weak granular bar under the claws of the first three pairs of legs.

Eggs ornamented, 58.1–68.1 μm in diameter excluding processes, and laid free (Figs. 5, 6, Additional file 15: Figure S12). Egg processes, 15–19 per hemisphere, in the shape of relatively long cones with slightly jagged apices. Processes reticulated, but only slightly scaled. Five tiles, each one subdivided in two parts by large septa, around the process base.

*Other material from the same sample*: paratypes similar to the holotype. Males and females present. Six bivalents in the oocytes. Paratypes morphometric data are reported in Table 2.

*Type locality*: Piane di Mocogno (Modena), Italy (sample C2112; 44°N 16.775;, 010°E 40.133).

*Type repositories*: the holotype (slide C2112-S10), 16 paratypes and nine eggs are mounted on slides in Faure Berlese mounting medium and deposited in the Bertolani collection at the Department of Life Sciences, University of Modena and Reggio Emilia, Italy, two paratypes are deposited in the Museum of Natural History of Verona, Italy.

*Differential diagnosis*: *Paramacrobiotus celsus* sp. n. differs from the other species of the *P. richtersi* complex in the egg shape and with *P. fairbanksi* even in the sex condition. The granulation on the hind legs is more evident than that of *P. spatialis* sp. n. and *P. depressus* sp. n. The egg processes are higher in elevation and in lower number with respect to those of the other species here described, apart *P. richtersi*, but they differ from the processes of this last species because they are not shaped like an inverted funnel.

*Paramacrobiotus celsus* sp. n. has macroplacoids and no eyespots, so it differs from all the *Paramacrobiotus* species without microplacoid and/or with eye spots, and it differs from the species with microplacoid and without eye spots for the following characters: *-P. alekseevi*: Animals: for smaller latero-ventral transversal crests without denticulate anterior margins and one ventral median crest not subdivided in smaller pieces in the buccal armature, absence of small teeth in the hind lunules, and presence of a fine granulation on the first three pairs of legs. *-P. chieregoi*: Animals: ventral median crest not subdivided in smaller pieces in the buccal armature. Eggs: for shorter egg processes (about 28 μm in height in *P*. *chieregoi*) of more regular conical shape, and for the presence of tiles around the process base. *-P. danielisae*: Animals: for the absence of fine sculpture of very small polygons on the cuticle surface. Eggs: for shorther processes. *-P. garynahi*: Animals: for the absence of oval pores on the cuticle surface, and median ventral crest subdivided in smaller pieces in the buccal armature. Eggs: for shorter egg processes without cap-like structures at their apices. *-P. gerlachae*: Animals: for an evident granulation in the first three pairs of legs. Eggs: for longer processes with slightly jagged apices. *-P. halei*: Animals: for the absence of very small tubercles on the cuticle surface. Eggs: for longer processes. *-P. hapukuensis*: Animal: for the presence of granulations in all pairs of legs. Eggs: for the shape of processes without a finger-shaped terminal portion. *-P. lorenae*: Eggs: for the shape of processes without a finger-shaped terminal portion, and an evident reticulated surface (with LM). *-P. peteri*: Animal: for the presence of granulations in all pairs of legs. Eggs: for the larger processes, without apices subdivided into a number of points, and for the larger number of areolae around each process (6–7 in *P. peteri*).

### *Paramacrobiotus arduus* sp. n. (Figs. 5, 7, 8, Additional file 16: Figure S13, Additional 17: Figure S14)

ZooBank registration: urn:lsid:zoobank.org:act:777D811F-A5E2-4D8B-83C2-CDF0EB7F03A3.

*Holotype*. slide C2703–1. Prodo (Terni), Italy; 42°N 45.853, 012°E 13.476.

*Paratypes*. Same data as for holotype,

*Etymology. arduus* (latin), “steep”, referring to the shape of the egg processes, but also “difficult” to the difficulty of morphologically recognizing the species.

#### Description

*Holotype*. Animal length 463.0 μm (morphometric data in Table 2, Additional file 1: Table S3). Lactescent. Sex undetermined. Without eyes. Cuticle smooth, without pores. Granulation on the surface of the hind legs spread, not so dark in PhC, slightly evident in the three first pairs of legs. Mouth surrounded by wide square peribuccal lamellae. Buccal armature (Additional file 16: Figure S13B, C): anterior band at the beginning of the buccal ring (at the base of the buccal lamellae) with a band of small teeth; posterior band at the beginning of the buccal tube (caudal part of the mouth) with a round row of large cusped teeth, sometimes jointed two by two, similar dorsally and ventrally, followed by three transversal crests, shorter on the ventral side. Median ventral crest triangular. Buccal tube length 44.0 μm, internal width 11.7 μm (*pt* 26.6). Insertion of the stylet supports at 36.6 μm (*pt* 83.2) from the beginning of the buccal tube. Pharyngeal bulb with large apophyses, three rod-shaped macroplacoids, the third with an evident terminal constriction, and a microplacoid (Additional file 16: Figure S13B, C). Macroplacoid row length 25.5 μm (*pt* 58.0), first macroplacoid 8.1 μm (*pt* 18.4), second macroplacoid 7.5 μm (*pt* 17.0), third macroplacoid 9.6 μm (*pt* 21.8) in length. Big seed-shaped microplacoid (3.5 μm in length), far from the macroplacoids and not in line with them, but parallel to the buccal tube. Claws of *hufelandi* type, with smooth lunules at their base, larger in the hind legs (Additional file 16: Figure S13D, E). External claw in the second pairs of legs 11.2 μm in length (*pt* 25.5); posterior claw of the hind legs 13.1 μm in length (*pt* 29.8). A weak granular bar present under the claws of the first three pairs of legs.

*Other material from the same sample*: paratypes similar to the holotype. Males and females present. Six bivalents in the oocytes. Paratypes morphometric data are referred in Table 2.

Eggs ornamented, 55.3–62.3 μm in diameter excluding processes, and laid free (Figs. 5, 7, 8d, e, Additional file 17: Figure S14): Egg processes, 16–21 per hemisphere, as truncated cones (12.1–18.3 μm in height, 10.4–16.3 μm in diameter), with a fine and often homogeneous reticulation and with a narrow top. The base of the processes surrounded by five pairs of large tiles (or five clearly subdivided in two), with pits on their bottom (with LM), which look very clear.

*Type locality*: Prodo (Terni), Italy (sample C2703; 42°N 45.853;, 012°E 13.476, 470 m a.s.l.).

*Type repositories*: the holotype (slide C2703-S1), 16 paratypes and 9 eggs are mounted on slides in Faure Berlese mounting medium and deposited in the Bertolani collection at the Department of Life Sciences, University of Modena and Reggio Emilia, Italy, 1 paratype is deposited in the Museum of Natural History of Verona, Italy.

*Differential diagnosis*: This species differs from *P. richtersi* in the shape of the eggs: the egg processes are not funnel-like and are clearly lower in height. The peculiarly narrow apex of the processes distinguishes *P. arduus* sp. n. from the other species of the *P. richtersi* complex here described. *Paramacrobiotus arduus* sp. n. differs from *P. spatialis* sp. n. for the narrower areolation as well, from *P. celsus* sp. n. for having shorter processes, and from *P. depressus* sp. n. due to higher processes. Regarding the animals, *P. arduus* sp. n. seems to have the less contrasting granulation on the legs. In addition, *P. arduus* sp. n. can be distinguished by *P. fairbanksi* due to the presence of males absent in the latter species.

*Paramacrobiotus arduus* sp. n. has macroplacoids and no eye spots, so it differs from all the *Paramacrobiotus* species without microplacoid and/or with eye spots, and it differs from the species with microplacoid and without eye spots for the following characters: *-P. alekseevi*: Animals: for smaller latero-ventral transversal crests without denticulate anterior margins and median ventral crest not subdivided in smaller pieces in the buccal armature, and absence of small teeth in the hind lunules. Eggs: for more regular, cone-shaped, egg processes without cap-like vesicular structures on their apices. *-P. chieregoi*: Animals: ventral median crest not subdivided in smaller pieces in the buccal armature. Eggs: for shorter egg processes (about 28 μm in height in *P*. *chieregoi*) of more regular conical shape, and for the presence of tiles around the process base. *-P. danielisae*: Animals: for the absence of fine sculpture of very small polygons on the cuticle surface. Eggs: for shorther processes. *-P. garynahi*: Animals: for the absence of oval pores on the cuticle surface, and median ventral crest subdivided in smaller pieces in the buccal armature. Eggs: for shorter egg processes without cap-like structures at their apices. *-P. gerlachae*: Animals: for the presence of a triangular median ventral crest in the buccal armature, and for the presence of a granulation in the first three pairs of legs. Eggs: for the shape of the processes, as truncated cones with a narrow top, and for the shape of the areolae around processes, that are large and clearly subdivided in two by a septum connecting the processes. *-P. halei*: Animals: for the absence of very small tubercles on the cuticle surface. Eggs: for longer processes. *-P. hapukuensis*: Animal: for the presence of granulations in all pairs of legs. Eggs: for the shape of processes without a finger-shaped terminal portion.

*-P. lorenae*: Eggs: for the shape of processes without a finger-shaped terminal portion, and an evident reticulated surface (with LM). *-P. peteri*: Animal: for the presence of granulations in all pairs of legs. Eggs: for the larger processes, without apices subdivided into a number of points, and for the larger number of areolae around each process (6–7 in *P. peteri*).

*Remarks*: In the same sample (C2703) a second species, *Paramacrobiotus arduus* [Ca1 MK041022], was identified by molecular analyses but resulted morphologically indistinguishable from *P. arduus* sp. n.

## Additional files

Additional file 1: **Table S3.** Morphometric data of the specimens of the *Paramacrobiotus* populations. (XLSX 32 kb)

Additional file 2: **Table S1.** Genetic distances (p-distance) computed among and within populations. All new haplotypes and those available in GenBank have been considered in the analysis, which was carried out on a 606 bp dataset. (XLSX 19 kb)

Additional file 3: **Table S2.** Genetic distances (p-distance) computed among species of different eutardigrade genera which sequences are available in GenBank. (XLSX 11 kb)

Additional file 4: **Figure S1.** Eggs of *Paramacrobiotus richtersi*. - A, B. Egg surface. - C, D. Eggs processes (lateral view). - E. In toto. A, C DIC; B, D, E PhC. Bars: A-D = 10 μm, E = 20 μm. (JPG 1266 kb)

Additional file 5: **Figure S2.** *Paramacrobiotus fairbanksi* from Riccò (PhC). - A. Animal in toto. - B. Buccal-pharyngeal apparatus. - C. Buccal armature (dorsal view). - D. Buccal armature (ventral view). - E. Claws of the third pair of legs. - F. Claws of the fourth pair of legs. Bars: A = 50 μm, B-F = 10 μm. (JPG 1282 kb)

Additional file 6: **Figure S3.** Eggs of *Paramacrobiotus fairbanksi* populations (SEM). - A-D. Riccò. – E. Pondel. – F. Rocchetta. Bars: A-B, E-F = 5 μm, C-D = 2 μm. (JPG 4101 kb)

Additional file 7: **Figure S4.** Eggs of *Paramacrobiotus fairbanksi* from Riccò. - A-B, D. Egg surface. - C. Egg processes (lateral view). - E-F. In toto. A, C, E-F PhC; B, D DIC. Bars: A-D = 10 μm, E = 20 μm. (JPG 1343 kb)

Additional file 8: **Figure S5.** *Paramacrobiotus spatialis* sp. n., holotype (PhC). - A. In toto. - B. Buccal-pharyngeal apparatus. - C. Buccal armature (ventral view). - D. Buccal armature (dorsal view). - E-F. Claws of the third pair of legs in different focal planes. – G. Claws of the fourth pair of legs. Bars: A = 50 μm, B-G = 10 μm. (JPG 1043 kb)

Additional file 9: **Figure S6.** Eggs of *Paramacrobiotus spatialis* sp. n., paratypes. - A-B. Egg processes (lateral view). - C-D. Egg surface. - E. In toto. A, C PhC; B, D-E DIC. Bars: A-D = 10 μm, E = 20 μm. (JPG 1119 kb)

Additional file 10: **Figure S7.** Eggs of *Paramacrobiotus spatialis* [Ca1 Guidetti et al. 2018]. - A In toto (SEM). - B. Egg process (SEM). - C-D. Egg surface. - E-F Egg processes (lateral view). - G In toto. C, E, G PhC. D, F DIC. Bars: A = 5 μm, B = 2 μm, C-E = 10 μm, G = 20 μm. (JPG 4012 kb)

Additional file 11: **Figure S8** *Paramacrobiotus depressus* sp. n., holotype (PhC). - A. In toto. - B. Buccal-pharyngeal apparatus. - C. Mouth. - D-E Claws of the third pair of legs at different focuses. - F. Claws of the fourth pair of legs. Bars: A = 50 μm, B-G = 10 μm. (JPG 1553 kb)

Additional file 12: **Figure S9.** Eggs of *Paramacrobiotus depressus* sp. n., paratypes (SEM). - A. Egg surface. - B. Egg process. - C. Tiles (areolae) with pits on their ground. - D. Internal view of a broken process. Bars: A = 5 μm, B-D = 2 μm. (JPG 5760 kb)

Additional file 13: **Figure S10.** Eggs of *Paramacrobiotus depressus* sp. n., paratypes. - A-B, D. Egg processes (lateral view). - C. Egg surface. - E. In toto. A, C, E PhC. B, D DIC. Bars: A-D = 10 μm, E = 20 μm. (JPG 1229 kb)

Additional file 14: **Figure S11.** *Paramacrobiotus celsus* sp. n., holotype (PhC). - A. In toto. - B. Buccal-pharyngeal apparatus. - C. Buccal armature (ventral view). - D. Buccal armature (dorsal view). E- Claws of the third pair of legs. - F-G. Claws of the fourth pair of legs in different focal planes. Bars: A = 50 μm, B-G = 10 μm. (JPG 4975 kb)

Additional file 15: **Figure S12.** Egg of *Paramacrobiotus celsus* sp. n., paratypes. - A. In toto (SEM). - B. Egg process (SEM). - C-D, F. Egg processes (lateral view). - E. Egg surface. - F. In toto. C, E, G PhC. D, F DIC. Bars: A = 5 μm, B = 2 μm, C-F = 10 μm, G = 20 μm. (JPG 8244 kb)

Additional file 16: **Figure S13.** *Paramacrobiotus arduus* sp. n., holotype (PhC). - A. In toto. - B. Buccal-pharyngeal apparatus (ventral view). - C. Buccal-pharyngeal apparatus (dorsal view). - D. Claws of the third pair of legs. - E. Claws of the fourth pair of legs. Bars: A = 50 µm, B-G = 10 µm. (JPG 2470 kb)

Additional file 17: **Figure S14.** Eggs of *Paramacrobiotus arduus* sp. n., paratypes. - A-B. Egg processes (lateral view). - C-D. Egg surface. - E. In toto. A, C, E PhC. B, D DIC. Bars: A-D = 10 µm, E = 20 µm (JPG 3490 kb)
