# Supplementary figures and images for: High diversity in species, reproductive modes and distribution within the Paramacrobiotus richtersi complex (Eutardigrada, Macrobiotidae)
Source: Zoological Lett. 2019 Jan 3;5:1. doi: 10.1186/s40851-018-0113-z (PMC6317227; doi:10.1186/s40851-018-0113-z)

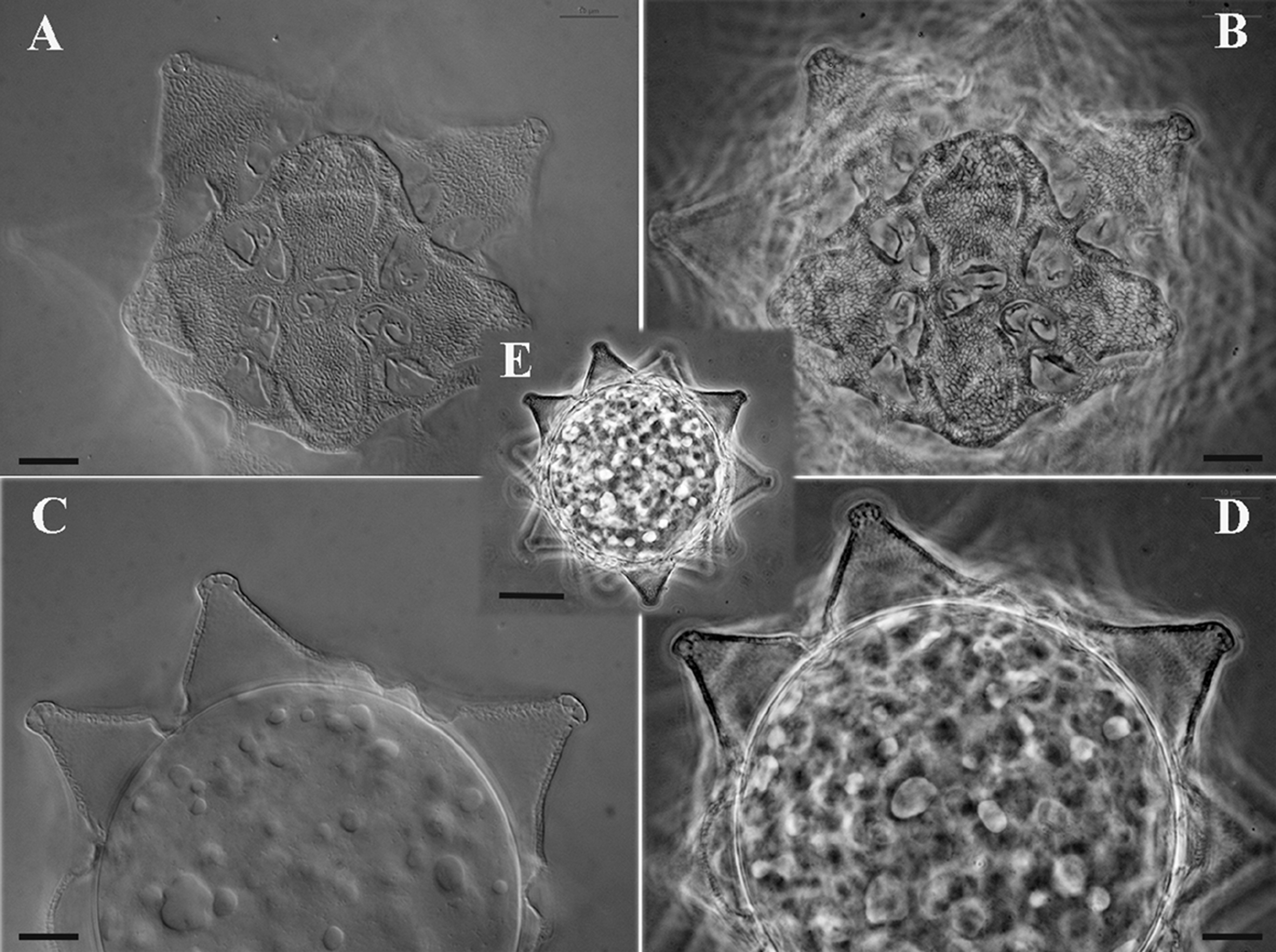

Supplement: Supplementary file 4 — Figure S1. Eggs of Paramacrobiotus richtersi. - A, B. Egg surface. - C, D. Eggs processes (lateral view). - E. In toto. A, C DIC; B, D, E PhC. Bars: A-D = 10 μm, E = 20 μm. (JPG 1266 kb) [file 40851_2018_113_MOESM4_ESM.jpg]

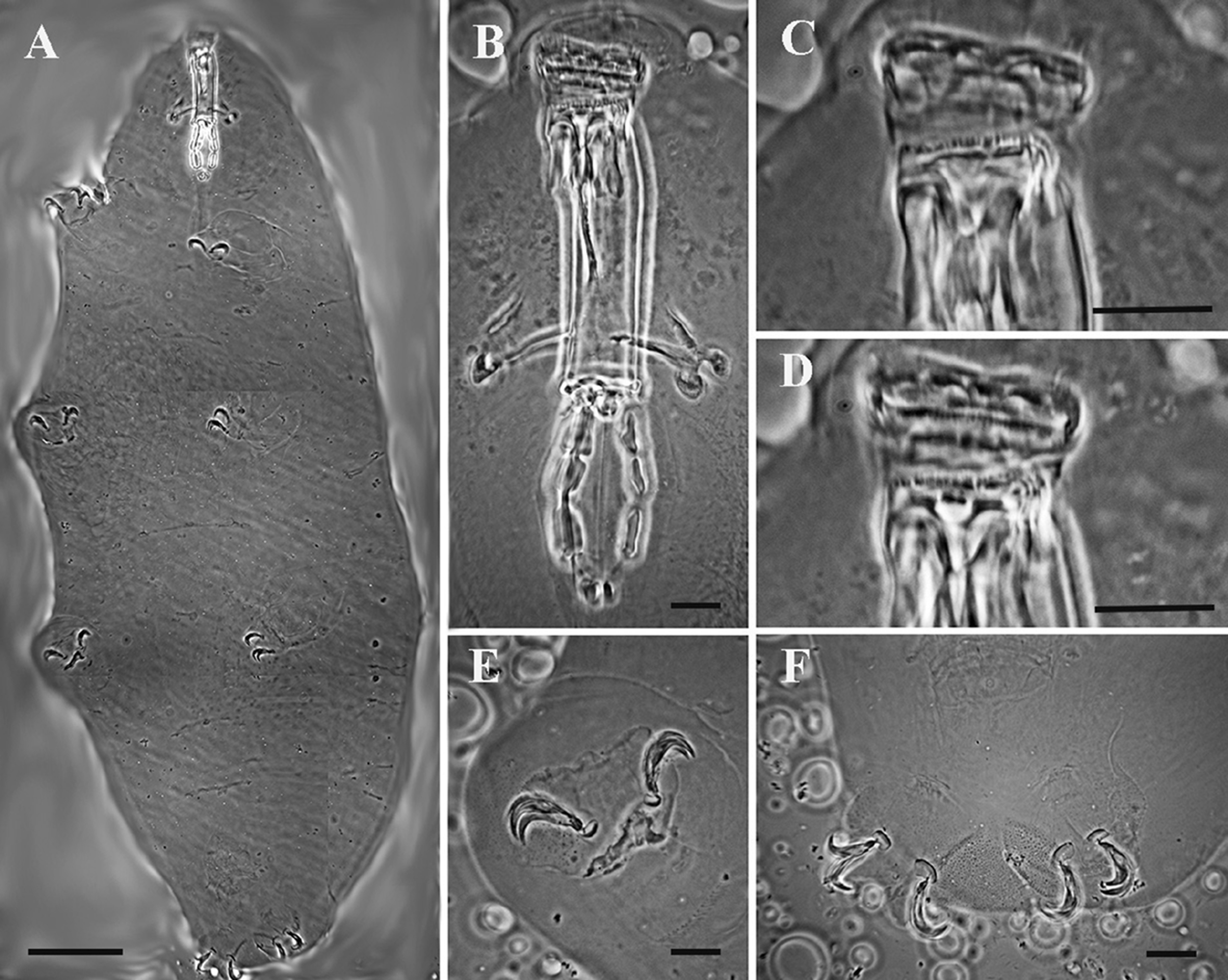

Supplement: Supplementary file 5 — Figure S2. Paramacrobiotus fairbanksi from Riccò (PhC). - A. Animal in toto. - B. Buccal-pharyngeal apparatus. - C. Buccal armature (dorsal view). - D. Buccal armature (ventral view). - E. Claws of the third pair of legs. - F. Claws of the fourth pair of legs. Bars: A = 50 μm, B-F = 10 μm. (JPG 1282 kb) [file 40851_2018_113_MOESM5_ESM.jpg]

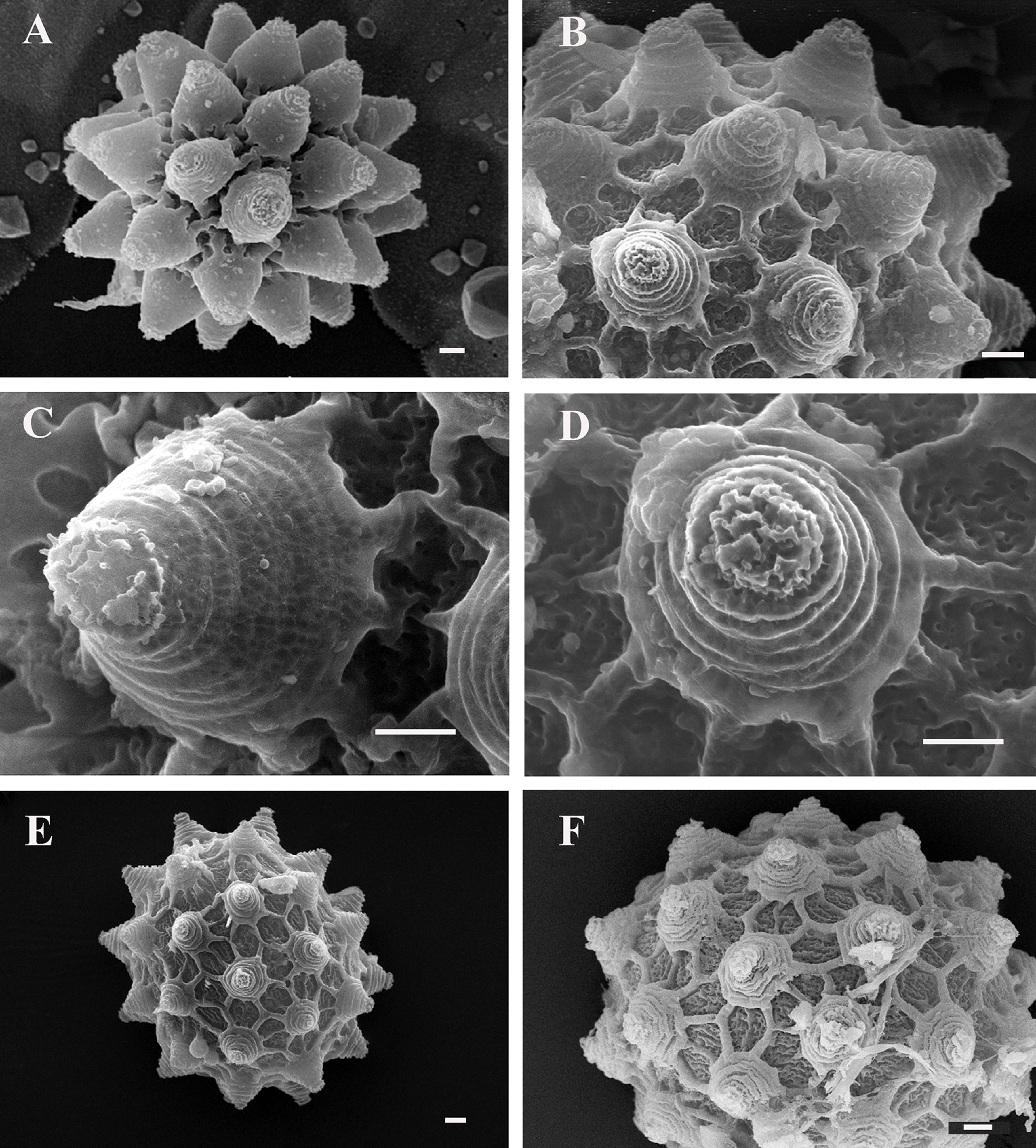

Supplement: Supplementary file 6 — Figure S3. Eggs of Paramacrobiotus fairbanksi populations (SEM). - A-D. Riccò. – E. Pondel. – F. Rocchetta. Bars: A-B, E-F = 5 μm, C-D = 2 μm. (JPG 4101 kb) [file 40851_2018_113_MOESM6_ESM.jpg]

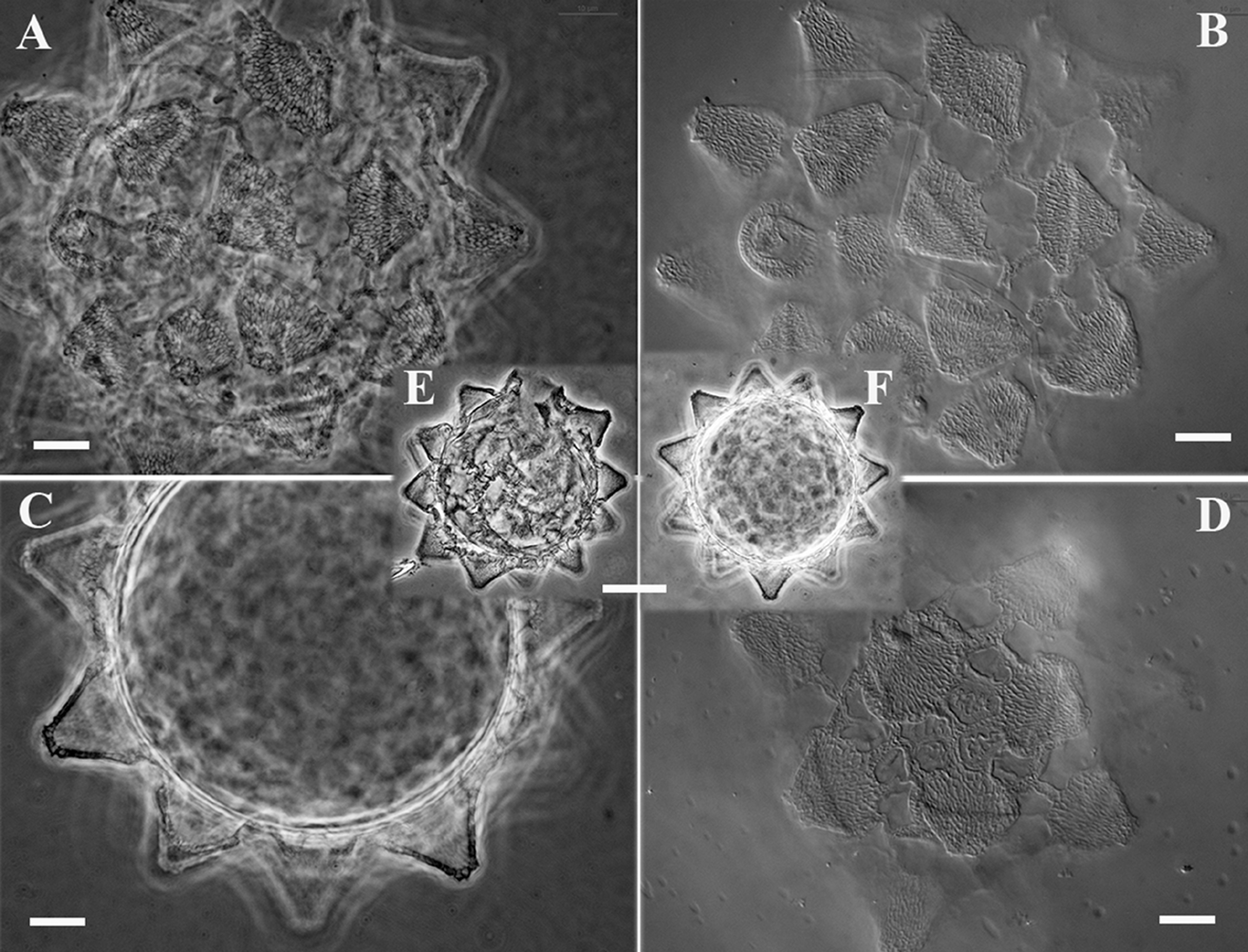

Supplement: Supplementary file 7 — Figure S4. Eggs of Paramacrobiotus fairbanksi from Riccò. - A-B, D. Egg surface. - C. Egg processes (lateral view). - E-F. In toto. A, C, E-F PhC; B, D DIC. Bars: A-D = 10 μm, E = 20 μm. (JPG 1343 kb) [file 40851_2018_113_MOESM7_ESM.jpg]

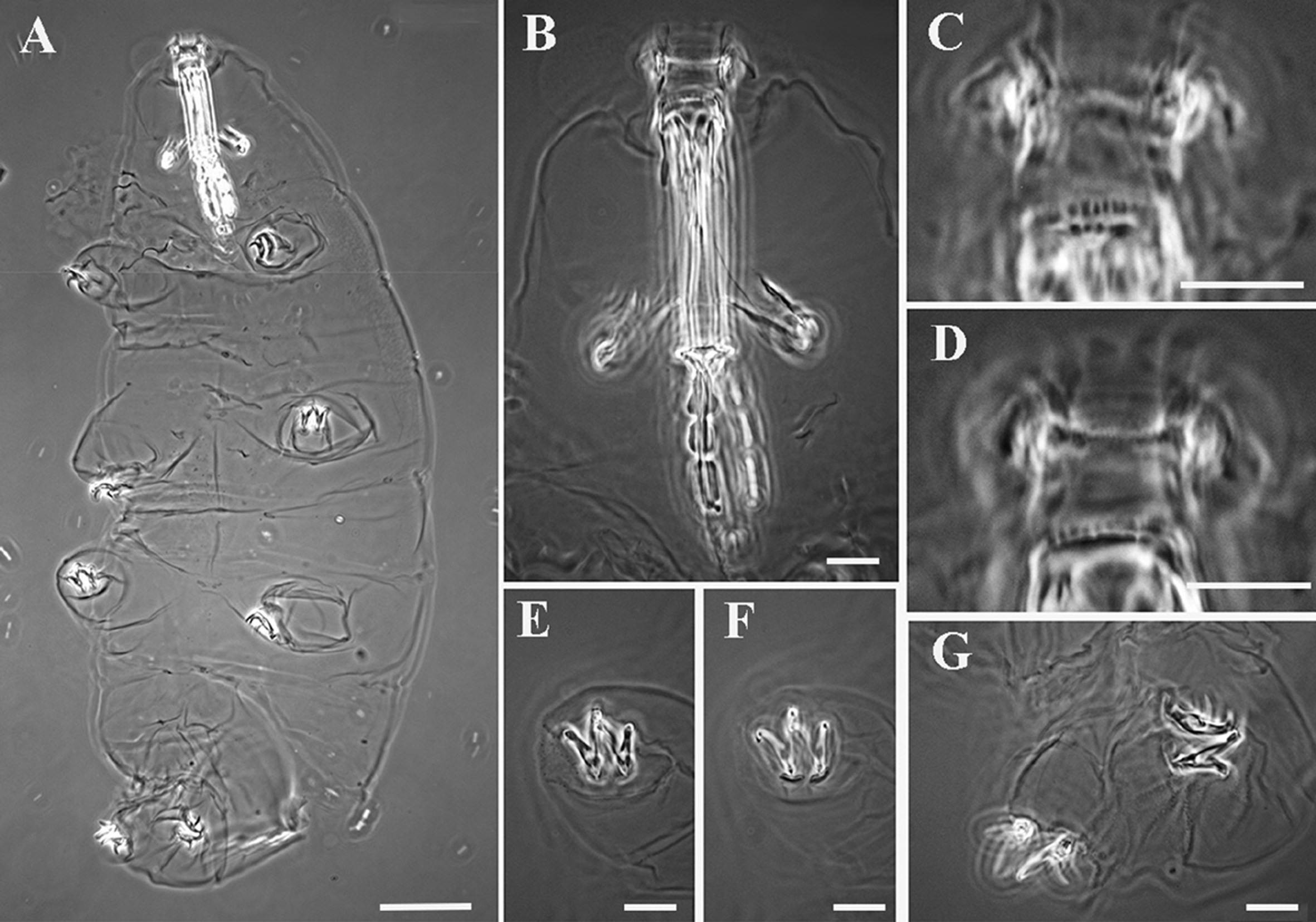

Supplement: Supplementary file 8 — Figure S5. Paramacrobiotus spatialis sp. n., holotype (PhC). - A. In toto. - B. Buccal-pharyngeal apparatus. - C. Buccal armature (ventral view). - D. Buccal armature (dorsal view). - E-F. Claws of the third pair of legs in different focal planes. – G. Claws of the fourth pair of legs. Bars: A = 50 μm, B-G = 10 μm. (JPG 1043 kb) [file 40851_2018_113_MOESM8_ESM.jpg]

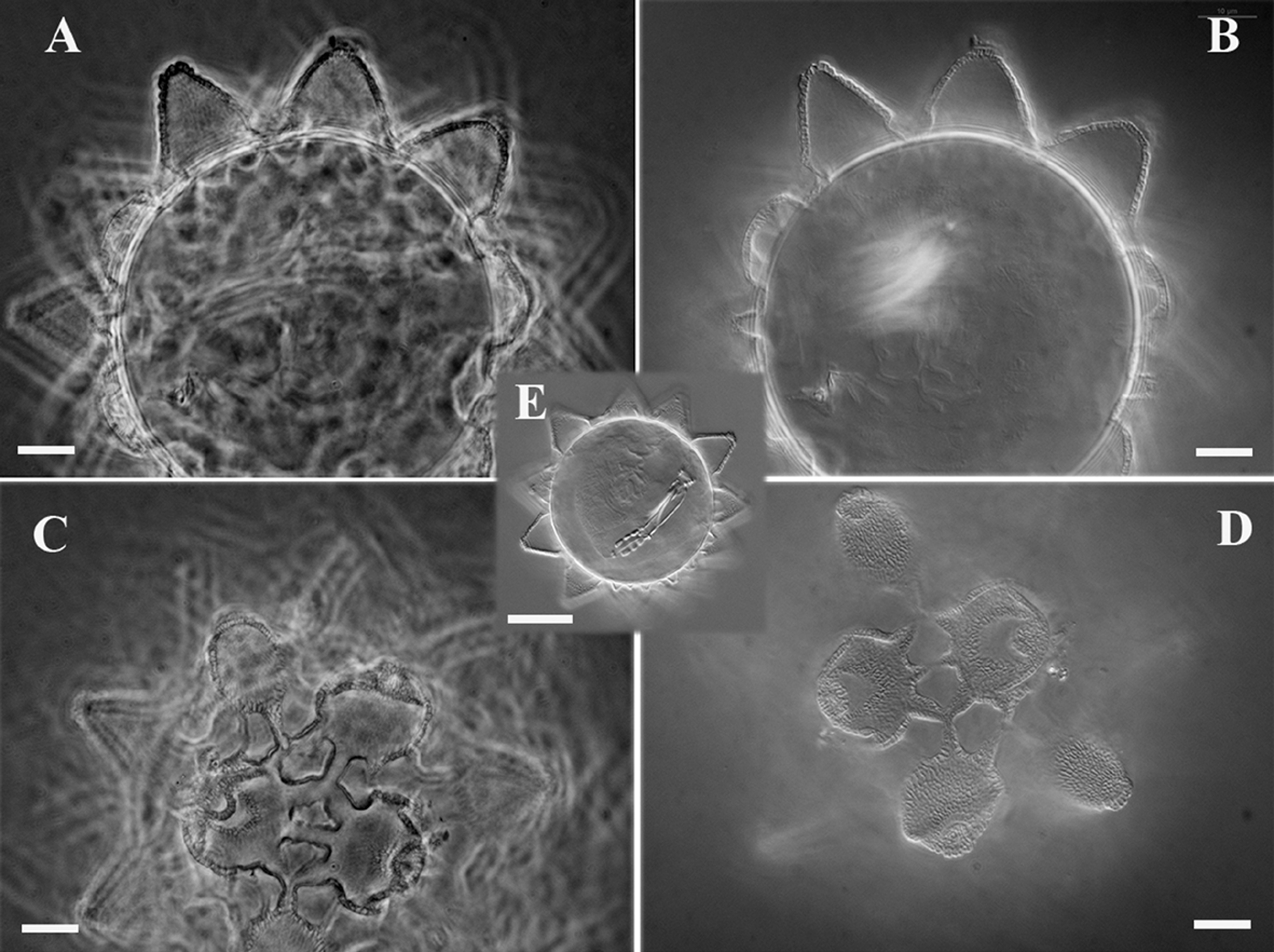

Supplement: Supplementary file 9 — Figure S6. Eggs of Paramacrobiotus spatialis sp. n., paratypes. - A-B. Egg processes (lateral view). - C-D. Egg surface. - E. In toto. A, C PhC; B, D-E DIC. Bars: A-D = 10 μm, E = 20 μm. (JPG 1119 kb) [file 40851_2018_113_MOESM9_ESM.jpg]

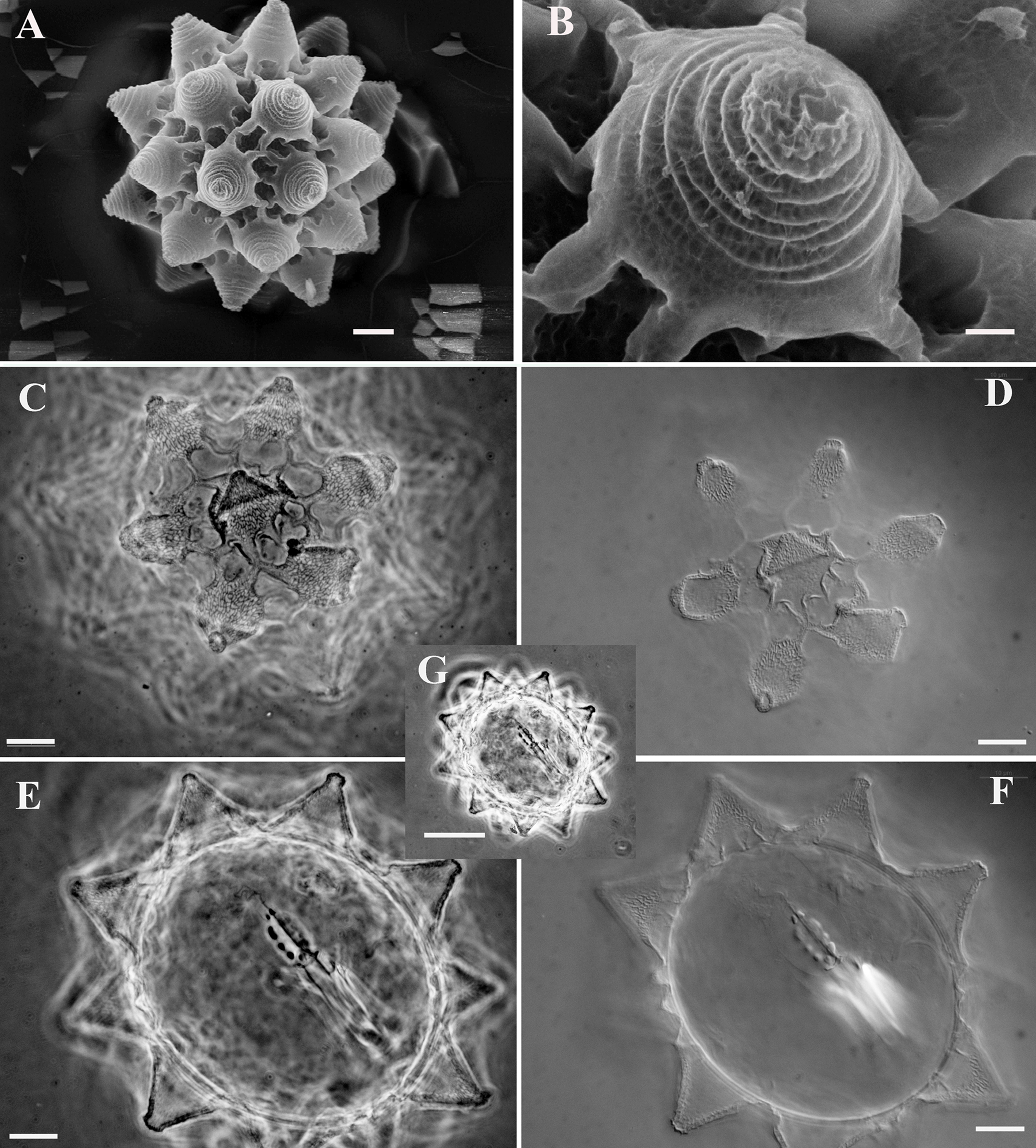

Supplement: Supplementary file 10 — Figure S7. Eggs of Paramacrobiotus spatialis [Ca1 Guidetti et al. 2018]. - A In toto (SEM). - B. Egg process (SEM). - C-D. Egg surface. - E-F Egg processes (lateral view). - G In toto. C, E, G PhC. D, F DIC. Bars: A = 5 μm, B = 2 μm, C-E = 10 μm, G = 20 μm. (JPG 4012 kb) [file 40851_2018_113_MOESM10_ESM.jpg]

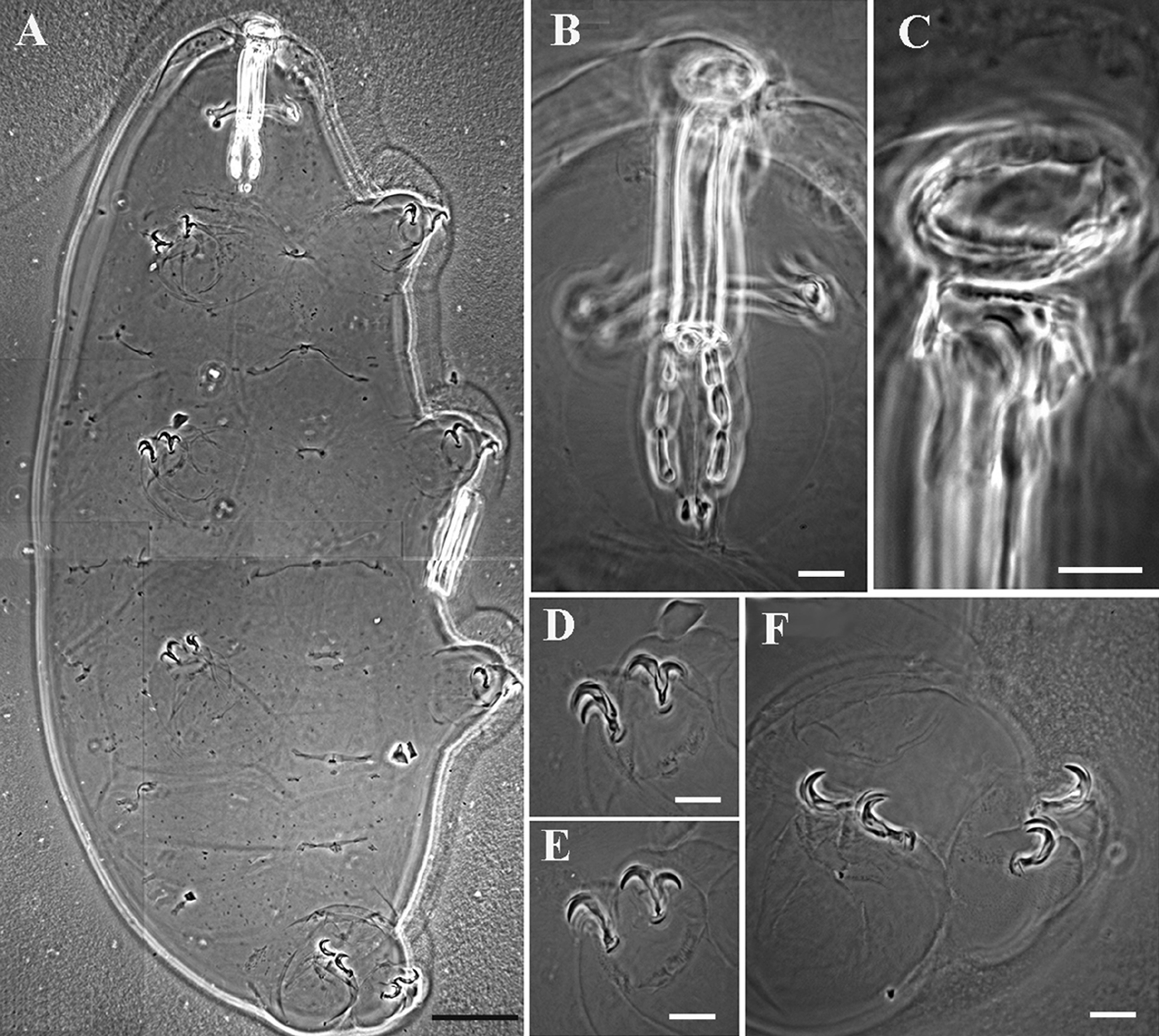

Supplement: Supplementary file 11 — Figure S8 Paramacrobiotus depressus sp. n., holotype (PhC). - A. In toto. - B. Buccal-pharyngeal apparatus. - C. Mouth. - D-E Claws of the third pair of legs at different focuses. - F. Claws of the fourth pair of legs. Bars: A = 50 μm, B-G = 10 μm. (JPG 1553 kb) [file 40851_2018_113_MOESM11_ESM.jpg]

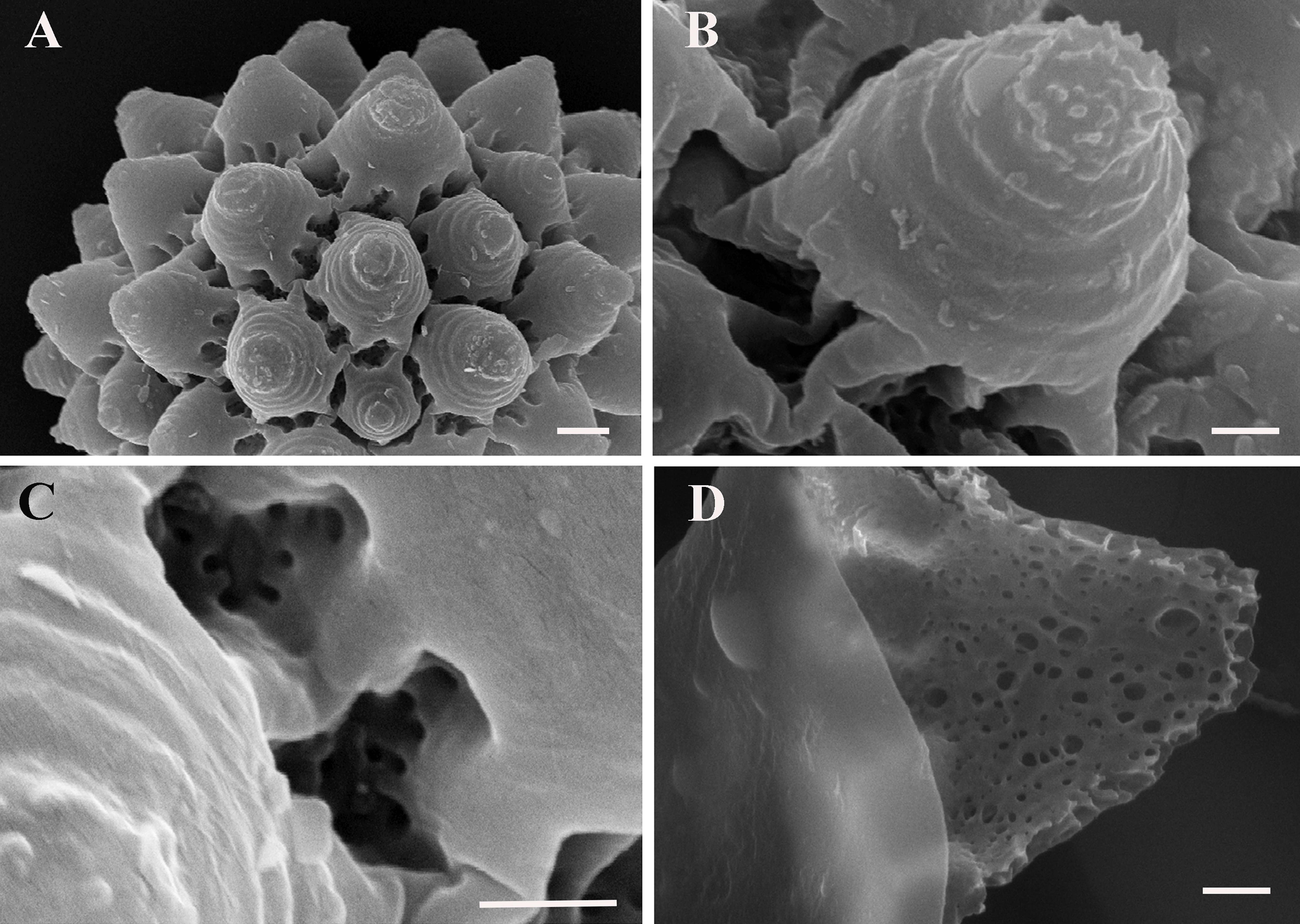

Supplement: Supplementary file 12 — Figure S9. Eggs of Paramacrobiotus depressus sp. n., paratypes (SEM). - A. Egg surface. - B. Egg process. - C. Tiles (areolae) with pits on their ground. - D. Internal view of a broken process. Bars: A = 5 μm, B-D = 2 μm. (JPG 5760 kb) [file 40851_2018_113_MOESM12_ESM.jpg]

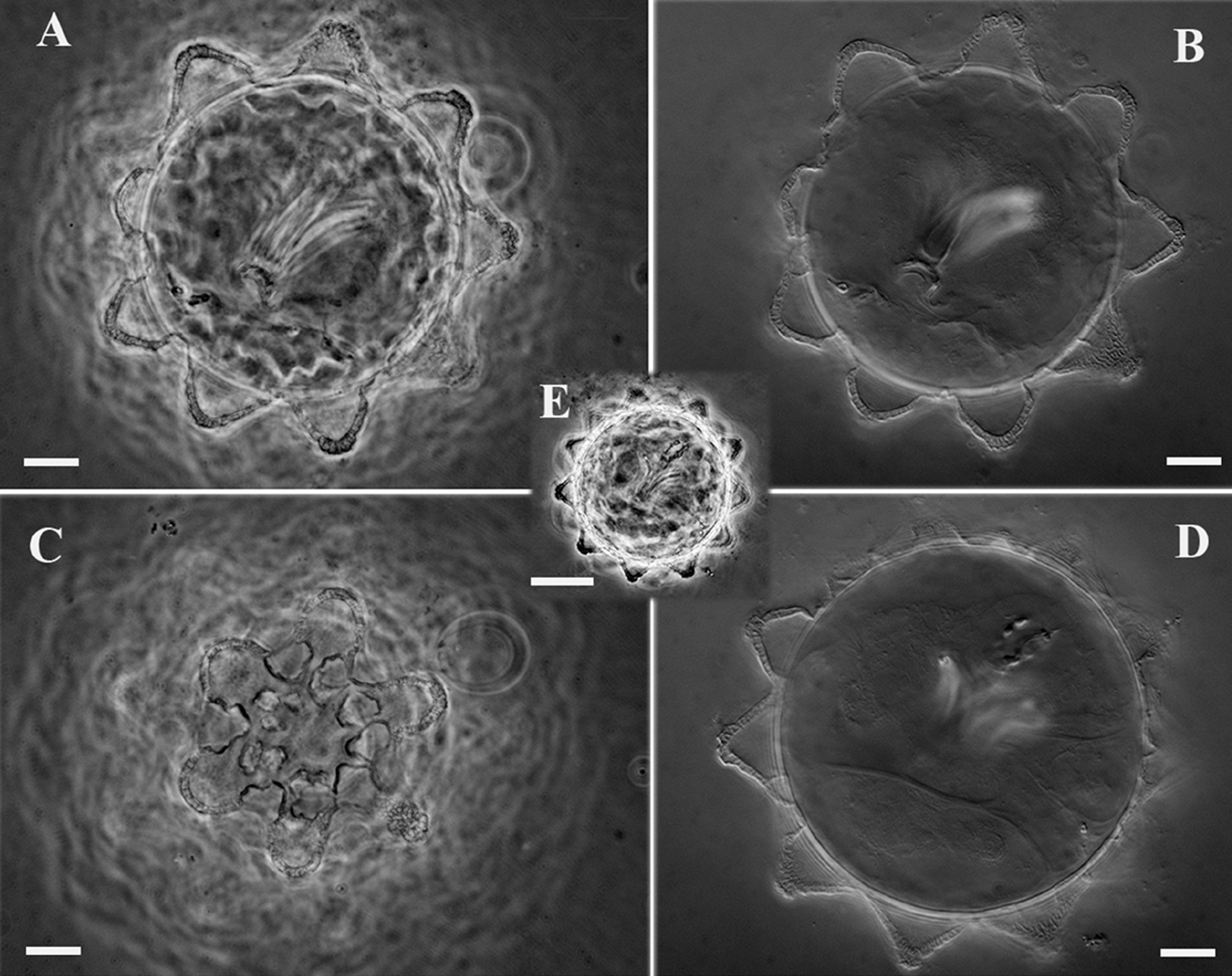

Supplement: Supplementary file 13 — Figure S10. Eggs of Paramacrobiotus depressus sp. n., paratypes. - A-B, D. Egg processes (lateral view). - C. Egg surface. - E. In toto. A, C, E PhC. B, D DIC. Bars: A-D = 10 μm, E = 20 μm. (JPG 1229 kb) [file 40851_2018_113_MOESM13_ESM.jpg]

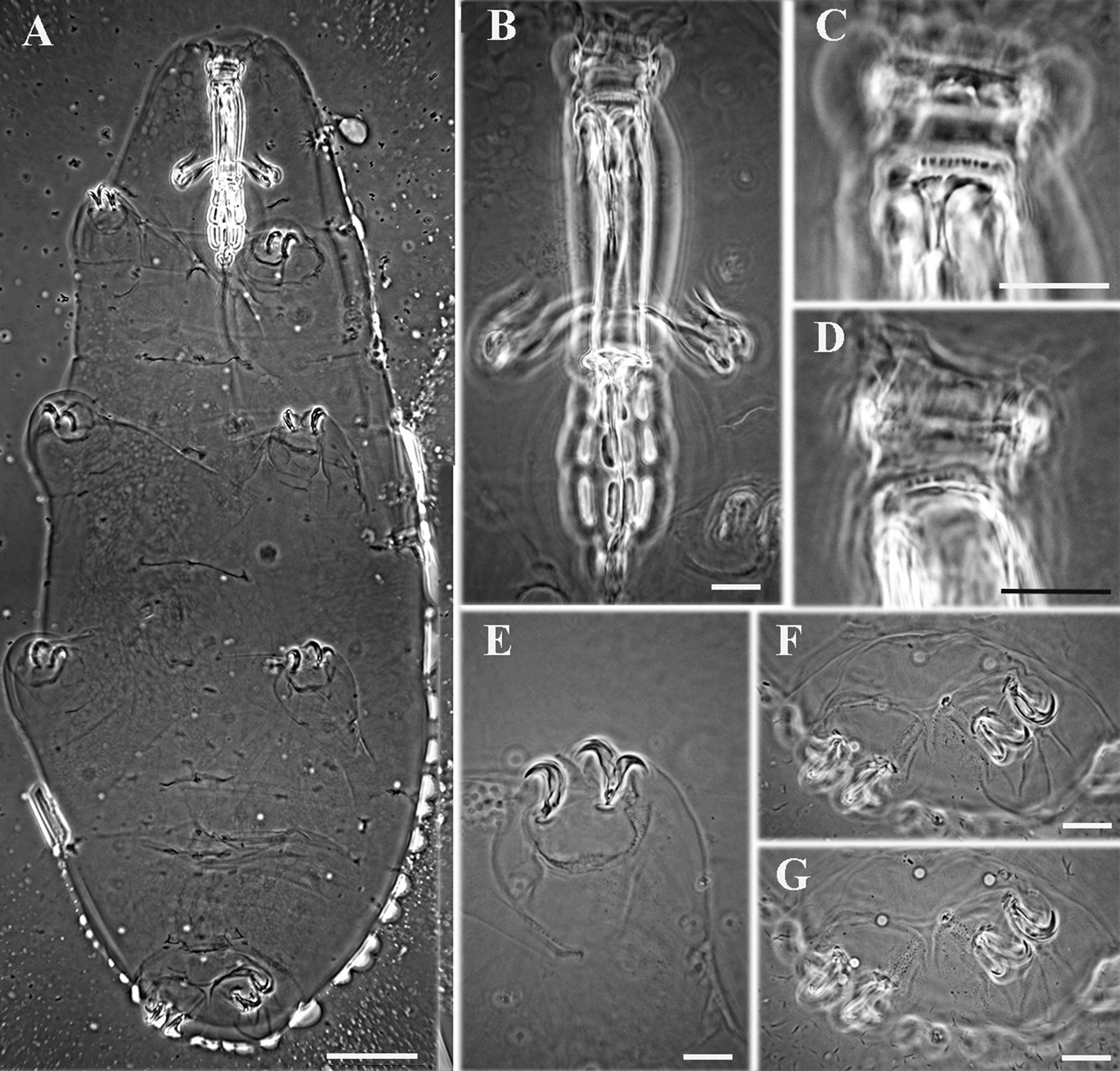

Supplement: Supplementary file 14 — Figure S11. Paramacrobiotus celsus sp. n., holotype (PhC). - A. In toto. - B. Buccal-pharyngeal apparatus. - C. Buccal armature (ventral view). - D. Buccal armature (dorsal view). E- Claws of the third pair of legs. - F-G. Claws of the fourth pair of legs in different focal planes. Bars: A = 50 μm, B-G = 10 μm. (JPG 4975 kb) [file 40851_2018_113_MOESM14_ESM.jpg]

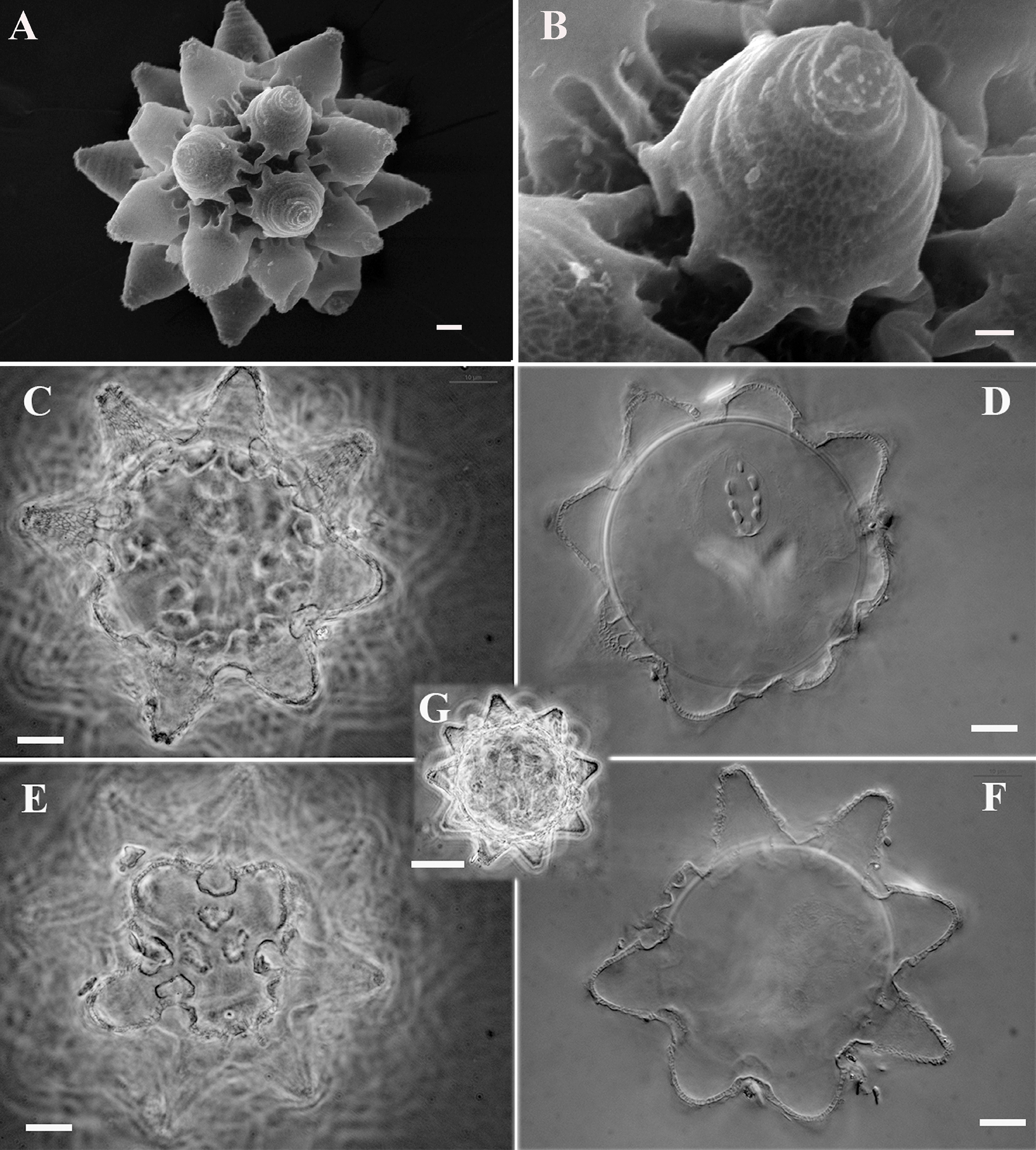

Supplement: Supplementary file 15 — Figure S12. Egg of Paramacrobiotus celsus sp. n., paratypes. - A. In toto (SEM). - B. Egg process (SEM). - C-D, F. Egg processes (lateral view). - E. Egg surface. - F. In toto. C, E, G PhC. D, F DIC. Bars: A = 5 μm, B = 2 μm, C-F = 10 μm, G = 20 μm. (JPG 8244 kb) [file 40851_2018_113_MOESM15_ESM.jpg]

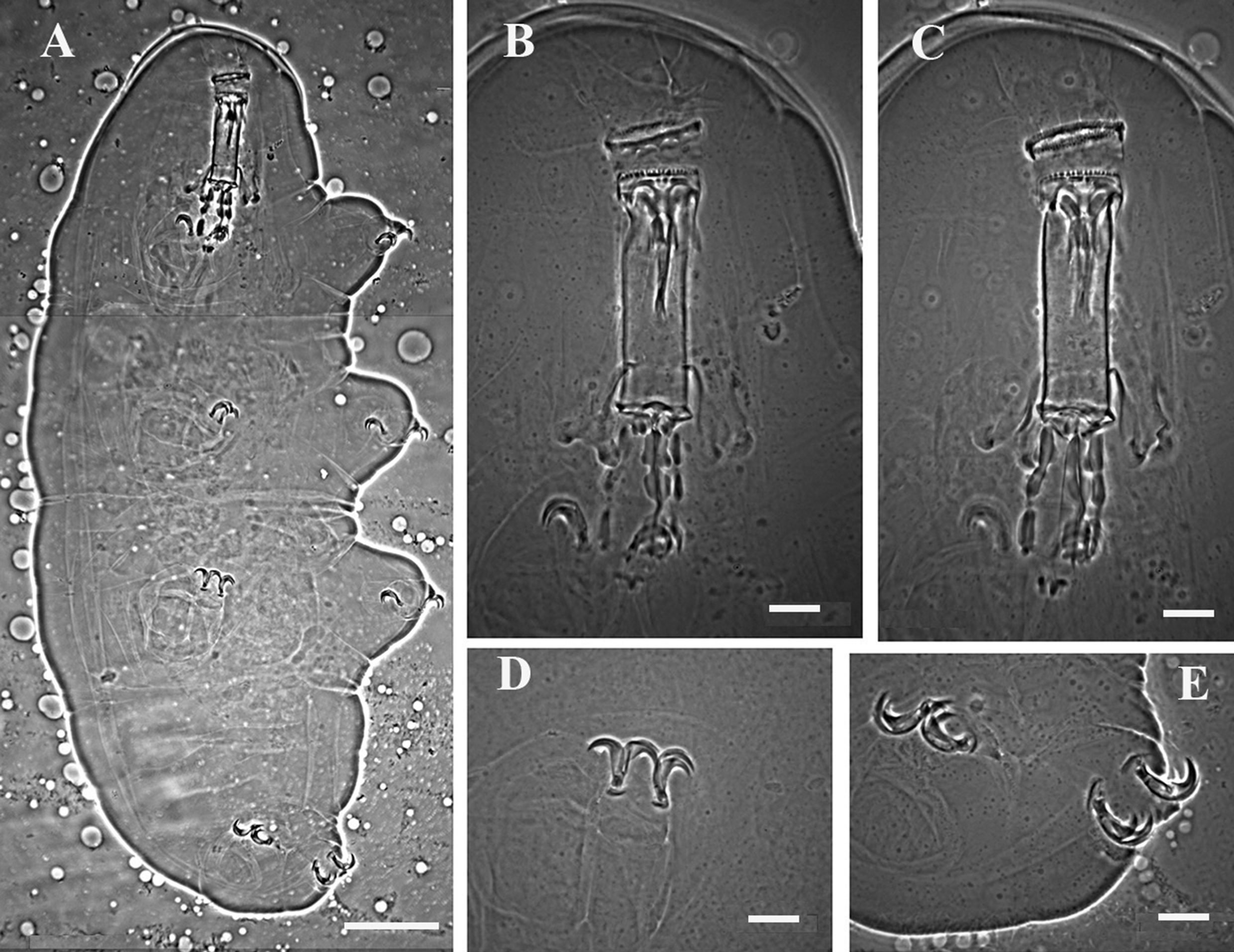

Supplement: Supplementary file 16 — Figure S13. Paramacrobiotus arduus sp. n., holotype (PhC). - A. In toto. - B. Buccal-pharyngeal apparatus (ventral view). - C. Buccal-pharyngeal apparatus (dorsal view). - D. Claws of the third pair of legs. - E. Claws of the fourth pair of legs. Bars: A = 50 µm, B-G = 10 µm. (JPG 2470 kb) [file 40851_2018_113_MOESM16_ESM.jpg]

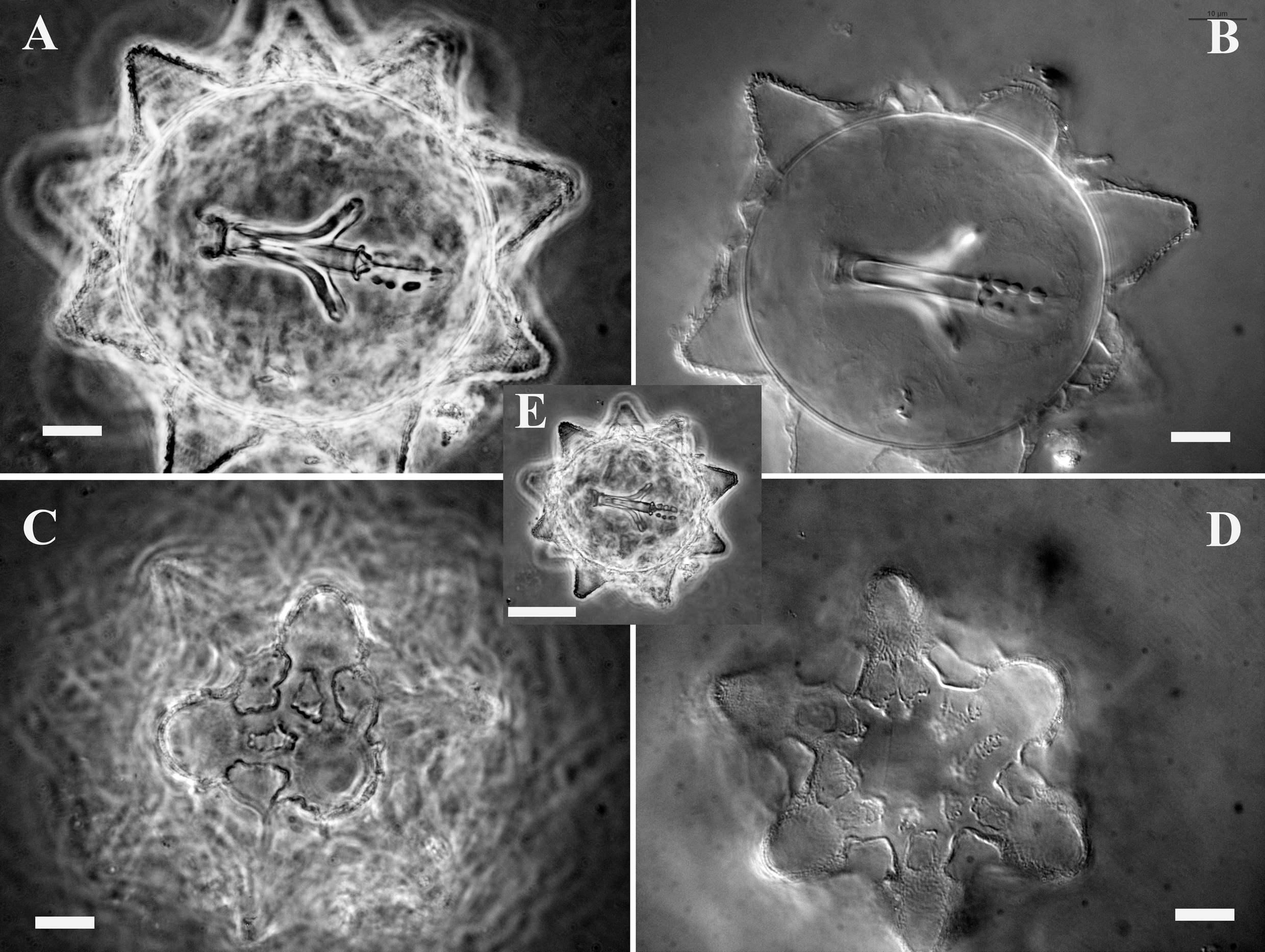

Supplement: Supplementary file 17 — Figure S14. Eggs of Paramacrobiotus arduus sp. n., paratypes. - A-B. Egg processes (lateral view). - C-D. Egg surface. - E. In toto. A, C, E PhC. B, D DIC. Bars: A-D = 10 µm, E = 20 µm (JPG 3490 kb) [file 40851_2018_113_MOESM17_ESM.jpg]
